# Enhancer Reprogramming Reveals the Tumorigenic Role of PTPRZ1 in Lung Squamous Cell Carcinoma

**DOI:** 10.1002/advs.202509344

**Published:** 2025-09-03

**Authors:** Yong‐Qiang Ning, Jie Wang, Yihui Zhang, Hongrui Li, Zenan Jiang, Xinyu Su, Xiao Yang, Xiaoxuan Wu, Bing Wei, Jianmin Ying, Jian Yan, Wenbin Li

**Affiliations:** ^1^ Department of Urology The First Affiliated Hospital of Xi'an Jiaotong University Xi'an 710061 China; ^2^ College of Life Sciences Northwest University Xi'an 710069 China; ^3^ Department of Pathology National Cancer Center/National Clinical Research Center for Cancer/Cancer Hospital Chinese Academy of Medical Sciences and Peking Union Medical College Beijing 100021 China; ^4^ Department of Molecular Pathology The Affiliated Cancer Hospital of Zhengzhou University & Henan Cancer Hospital Zhengzhou 450008 China; ^5^ Tung Biomedical Sciences Centre Department of Biomedical Sciences College of Biomedicine City University of Hong Kong Kowloon, Hong Kong Special Administrative Region China; ^6^ Department of Precision Diagnostic and Therapeutic Technology The City University of Hong Kong Shenzhen Research Institute Shenzhen 518016 China

**Keywords:** cancer epigenomics, lung squamous cell carcinoma, midkine, PTPRZ1, transcription factor

## Abstract

Lung squamous cell carcinoma (LUSC) remains a lethal malignancy with limited therapeutic advancements, underscoring the need to identify novel oncogenic drivers. This study integrates multi‐omics analyses—including ChIP‐seq, bulk RNA‐seq, single‐cell ATAC/RNA‐seq, and spatial transcriptomics—to delineate enhancer‐driven transcriptional networks in 59 matched LUSC tumors and adjacent normal tissues. The analyses identify 3,447 tumor‐specific oncogenic enhancers (SOEs) enriched for master transcription factors (SOX2, TP63, KLF5, GRHL2) that orchestrate malignant programs. Single‐cell epigenomic profiling reveals these SOEs to be exclusively active in a cancer cell‐like cluster, pinpointing PTPRZ1, a receptor tyrosine phosphatase, as a top SOE‐driven candidate. Functional studies demonstrate that PTPRZ1 is essential for LUSC‐tumorigenesis and tumor cells proliferation and migration in vitro and in vivo. Spatial transcriptomic analysis further implicates midkine (MDK) as the ligand activating PTPRZ1 in LUSC tumor tissue. Mechanistically, PTPRZ1 mediates MDK‐induced PI3K phosphorylation that is essential for LUSC tumor growth. This work illustrates enhancer addiction as a hallmark of LUSC, identifies the PTPRZ1‐MDK axis as an important targetable pathway, and establishes a paradigm for dissecting epigenetic vulnerabilities in solid tumors through multi‐omics integration. These findings advance precision oncology strategies for LUSC, bridging epigenomic dysregulation to therapeutic intervention.

## Graphical abstract

Lung squamous cell cancer (LUSC) is deadly due to lack of good treatments. Here, multiomics techniques are applied to LUSC tumors and find thousands of cancer‐specific enhancers controlling key genes. Interestingly, the gene PTPRZ1 is identified as a critical oncogene driven by the LUSC‐acquired enhancers. PTPRZ1 is activated by MDK, fueling cancer via the PI3K pathway, and blocking PTPRZ1 stopped cancer cell growth and spread, revealing PTPRZ1‐MDK as a new treatment target for LUSC.

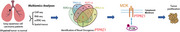

## 1 Introduction

Lung cancer remains the leading cause of cancer‐related mortality worldwide, accounting for ≈1.8 million deaths annually and representing 18.7% of global cancer fatalities.^[^ ^1^, ^2^ ^]^ Non‐small cell lung cancer (NSCLC) constitutes 85% of cases, with lung squamous cell carcinoma (LUSC) representing 20–30% subtypes.^[^ ^3^ ^]^ LUSC arises from bronchial epithelial cells and is tightly linked to smoking and environmental carcinogens.^[^ ^4^ ^]^ Unlike lung adenocarcinoma (LUAD), where targeted therapies confer significant clinical benefit, the efficacy of first‐line targeted therapies in LUSC remains limited. The low mutation frequency (<5%) in established driver genes (e.g., *EGFR*, *ALK*, *ROS1*, *MET*, *RET*, *HER2*) restricts the proportion of patients achieving clinical benefit. Furthermore, despite the high prevalence of recurrent genetic alterations — including mutations in *NFE2L2*, *KEAP1* and *PIK3CA* and amplifications of *SOX2*, *NSD3*, and *FGFR1* — clinically effective agents against these targets are still lacking.^[^ ^5^, ^6^, ^7^ ^]^ This underscores a critical unmet therapeutic need in LUSC patients.

Beyond genetic alterations, epigenetic dysregulation is increasingly recognized as a hallmark of tumorigenesis. The transient perturbation of Polycomb group protein‐mediated transcriptional silencing — sufficient to trigger an irreversible transition to a cancer cell fate in *Drosophila* —demonstrates that tumorigenesis can be initiated solely through non‐genetic mechanisms, independent of mutations.^[^ ^8^ ^]^ Hypermethylation of tumor suppressor promoters (e.g., *TP53*, *RB1*, *CDKN2A*) silences critical anti‐proliferative pathways, while histone modification enzymes like HDAC1 promote oncogenic phenotypes by repressing tumor suppressor genes (e.g., *TP53*) and enabling angiogenesis.^[^ ^9^, ^10^, ^11^ ^]^ Notably, epigenetic therapies, including HDAC inhibitors, have demonstrated preclinical efficacy in NSCLC models, yet their clinical translation in LUSC remains underexplored.^[^ ^12^ ^]^ Transcriptional reprogramming driven by enhancer plasticity shapes cancer progression. Enhancers and super‐enhancers (SEs) act as hubs for oncogenic transcription factors (TFs), such as SOX2 and p63 in squamous carcinomas, which establish lineage‐specific regulatory networks to activate genes like *KRT5* and *ETV4*.^[^ ^13^, ^14^, ^15^, ^16^, ^17^, ^18^ ^]^ These TFs cooperate with chromatin remodelers to prime accessible chromatin regions, enabling malignant transcriptional programs.^[^ ^19^, ^20^, ^21^ ^]^ While these findings underscore the significant role of transcriptional reprogramming in LUSC tumorigenesis, a comprehensive multi‐omics analysis of alterations in transcriptional regulatory network is still forthcoming. In addition, most studies rely on cell lines, which poorly recapitulate the epigenetic heterogeneity of primary tumors, leaving gaps in our understanding of in vivo enhancer dynamics in LUSC.^[^ ^14^, ^22^ ^]^

To address this, we integrated multi‐omics analyses of 59 matched‐pairs of primary LUSC tumors and non‐cancerous tissues. Using Chromatin Immunoprecipitation followed by Sequencing (ChIP‐seq) for genome‐wide profiling of histone H3 acetylated lysine 27 (H3K27ac hereafter) and mono‐methylated lysine 4 (H3K4me1 hereafter), we profiled LUSC‐specific enhancer landscape and linked them to transcriptional hubs governing proliferation and immune evasion. Combined with single‐cell Assay for Transposase‐Accessible Chromatin using sequencing (scATAC‐seq), our data revealed Receptor‐type tyrosine‐protein phosphatase zeta 1 (PTPRZ1), a receptor tyrosine phosphatase whose expression was predominantly restricted to the central nervous system (CNS) under physiological condition, as a novel LUSC‐enhancer‐driven oncogene. We functionally validated its oncogenic role through depletion and overexpression assays in cells and in mice. Subsequently, spatial transcriptomics analysis fine‐mapped that MDK could be the key ligand of PTPRZ1 in conferring its oncogenic activity in LUSC, suggesting a potential new therapeutic target in treating LUSC. Our work not only delineates the epigenetic basis of LUSC malignancy but also provides a framework for targeting enhancer‐addicted cancers.

## 2 Results

### 2.1 Epigenetic Remodeling has a Significant Contribution to the Tumorigenicity of LUSC

**Figure 1:**
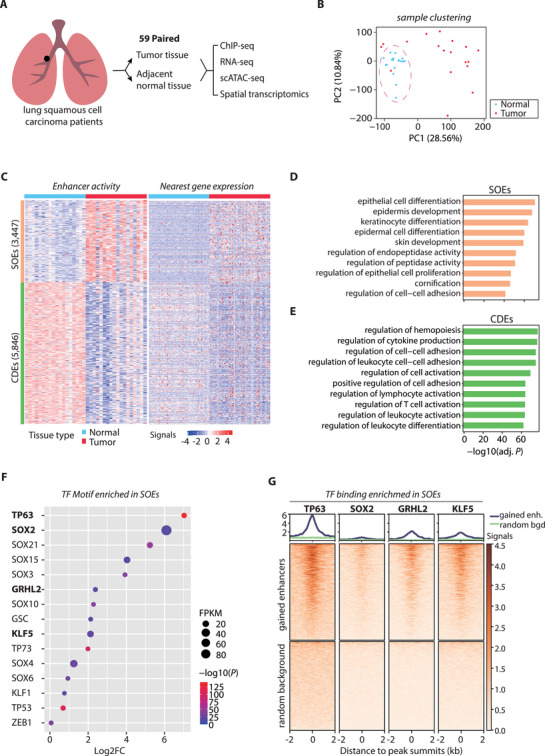
LUSC cells acquire oncogenic properties via epigenetic reprogramming. A) Schematic of the experimental workflow for analyzing enhancer landscapes in tumor and adjacent normal tissues from LUSC patients (ChIP‐seq: n = 18 matched pairs). B) Principal Component Analysis (PCA) of LUSC samples based on enhancer activity profiles (H3K27ac Reads Per Kilobase of transcript per Million mapped reads, also known as RPKM) demonstrates a clear separation between tumor (red) and normal (blue) tissues. Global epigenetic divergence between normal and tumor tissues was assessed using MANOVA, with significant results (Pillai's trace = 0.932, *p* < 0.001, partial η^2^ = 0.468). C) Heatmaps displaying the signal intensity of cancer gained enhancers (SOEs, n = 3447) and cancer depleted Enhancers (CDEs, n = 5846) using H3K27ac RPKM data from tumor and adjacent normal tissues of 18 LUSC patients (left). Corresponding heatmap for nearest gene expression profiles of SOEs and CDEs from 59 LUSC patients (right). D,E) Gene ontology (GO) analysis via GREAT analysis reveals the top 10 enriched terms for SOEs (D) and CDEs (E), ranked by fold enrichment with a significance threshold of FDR<0.05. F) *De novo* motif enrichment analysis of SOEs identifies LUSC lineage‐specific transcription factor (TF) binding motifs using Homer software (FDR < 0.01). The top enriched motifs and corresponding TFs are shown. The expression (Fragments Per Kilobase of transcript per Million mapped fragments, also known as FPKM) of the top 10 SOE‐associated TFs in tumor versus normal tissues (59 paired samples) is provided by the size of the dot. Significance (‐log10(P)) and fold change (Log2FC) from RNA‐seq results are also displayed according to the color key and position in X‐axis, respectively. G) Average plots and heatmaps of ChIP‐seq signals for squamous lineage TFs (TP63, SOX2, GRHL2, and KLF5) at SOE centers (±2 kb) compared to random genomic regions in HCC95 cells. Abbreviations: enh, enhancer; bgd, background.

We have collected tumors and adjacent normal tissues from 109 patients with LUSC in different clinical stages, of which 59 were paired biopsies from the same patients (**Figure** **1**A; Table S1A, Supporting Information). Epigenetic profiling was carried out for these samples, including ChIP‐seq on the active enhancer biomarkers, H3K4me1 and H3K27ac (Figure S1A,B, Supporting Information), as well as RNA‐seq. Principal component analysis (PCA) using either chromatin mark ChIP‐seq (Figure 1B) or RNA‐seq (Figure S1C, Supporting Information) alone could already well differentiate tumor and normal tissues (Multivariate Analysis of Variance, also known as MANOVA, *P* < 0.001), demonstrating the high‐quality of both datasets. It was also noted that tumors displayed high heterogeneity in terms of transcriptional program, unlike the normal samples that were clustered together in the PCA analysis.

To understand how the epigenetic changes influenced tumorigenesis, we identified putative enhancers by intersecting H3K4me1 and H3K27ac ChIP‐seq peaks which were located at least 2.5 kb away from any transcription start site (TSS) and were reproducibly present in at least two samples (Figure S1D, Supporting Information). When comparing the list of putative enhancers from tumor and adjacent normal tissues, we identified 3447 genomic elements that were specific to tumors (cancer‐gained) and we termed them as LUSC onco‐enhancers (SOE, hereafter) (Table S1B, Supporting Information). On the other hand, we also discovered 5846 putative enhancers that were common in normal tissues but missing from any of the tumor samples (cancer‐lost), which were termed cancer‐depleted enhancers (CDE, hereafter) (Figure 1C; Table S1C, Supporting Information). To test whether the analysis had comprehensively recovered all SOE and CDE, we conducted saturation analysis to detect the percentage of newly differential enhancers compared to the total differential enhancers as the number of analyzed samples increased, result showing that the vast majority (>95%) of SOE could be adequately identified with 6 sample pairs while CDE were also nearly saturated with just 10 pairs (Figure S1E, Supporting Information). Consistently, genes next to the SOE were upregulated in most of the 109 tumor samples compared to normal tissues, which included well‐known marker genes of LUSC such as SOX2, TP63 and NRF2 (Figure S2A,B, Supporting Information).^[^ ^23^ ^]^ The gene ontology (GO) analysis of SOE revealed that these tumor‐gained enhancer elements potentially regulated genes involved in epithelial cell differentiation, epidermis development, and keratinocyte differentiation, etc., corresponding to the typical characteristics of LUSC such as intercellular bridging, and keratinization of the individual cells (forming squamous pearls) (Figure 1D). In contrast, CDE‐adjacent genes generally exhibited weaker expression in cancers and were significantly implicated in regulation of immunity‐related pathways, including hemopoiesis, cytokine production and T cell activation (Figure 1E), suggesting that immunosuppression could be one of the key factors leading to development of LUSC.

Transcriptional program is primarily governed by sequence‐specific TFs.^[^ ^24^, ^25^ ^]^ Binding of key TFs, also known as master regulators, is usually enriched in cell fate determinant or disease‐specific *cis*‐regulatory elements, such as super‐enhancers.^[^ ^26^, ^27^ ^]^ To find out which TFs were essential for the tumorigenesis of LUSC, sequence motif enrichment analysis was performed in SOEs. As expected, the well‐established master TFs of LUSC, such as SOX2 and p63, exhibited the highest enrichment of binding sites among all tested TFs (Figure 1F). Besides TFs from the same structural families of these master TFs, which had near‐identical motifs such as multiple SOXs and p53/p73, significant enrichment of binding sites from TFs without a previously warranted role in LUSC were also detected, for instance GRHL2 and KLF5. It is interesting that both TFs have been annotated with an oncogenic role in other types of cancers.^[^ ^28^, ^29^, ^30^ ^]^ In particular, KLF5 has been shown in collaboration with SOX2 and p63 (coded by *TP63*) to drive core regulatory circuitry in esophageal squamous cell carcinoma (ESCC) cells, while GRHL2 and p63 mutually regulate each other to maintain the epithelial phenotype and plasticity, indicating their potential role in driving the tumorigenesis of squamous cell carcinoma.^[^ ^19^, ^31^ ^]^ To confirm their binding in SOEs, we performed ChIP‐seq for these factors respectively, including p63, SOX2, GRHL2 and KLF5, all of which exhibited significant and specific binding to most SOEs, compared to other non‐SOE genomic regions (Figure 1G).

### 2.2 Master TFs Shape the Epigenetic Landscape in Tumor Cell Cluster

**Figure 2:**
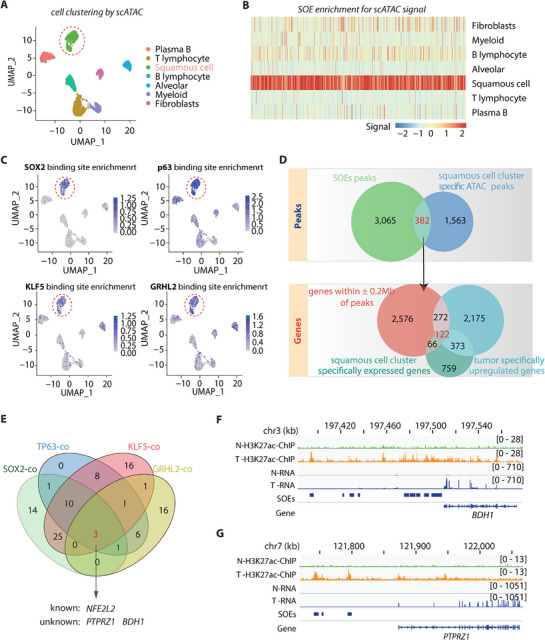
Influence of Master Transcription Factors on the Epigenetic Landscape in Tumor Cell Clusters. A) UMAP visualization of scATAC‐seq cell clusters from 19556 cells obtained from two LUSC patients, with dotted lines indicating squamous cell populations. B) Heatmap illustrating the Z‐score normalized chromatin accessibility of SOE (n = 3447) across various cell types as determined by scATAC‐seq. C) UMAP plots showcasing the enrichment scores of TF binding motifs for TP63, SOX2, GRHL2, and KLF5 across seven major cell types, with squamous cells outlined by dotted lines. D) The top Venn diagram displays the number of common peaks identified by intersecting the list of SOEs (n = 3447) with squamous cell cluster‐specific ATAC peaks (n = 1945), revealing 382 genomic fragments likely to be LUSC‐specific chromatin accessible loci. The lower section lists protein‐coding genes located within 200 kb of these loci (n = 3036), including 122 genes exhibiting specific upregulation in both bulk tumors (n = 2942) and squamous cell clusters (n = 1320) compared to adjacent normal tissues and other cell clusters within tumor tissues, respetively (Adjusted *P* < 0.05, log2FC > 1). E) Venn diagram showing the intersection of genes co‐expressed with TP63, SOX2, GRHL2, and KLF5 (Adjusted *P* < 0.05, r > 0.5). F,G) Normalized ChIP‐seq and RNA‐seq meta tracks depicting H3K27ac and mRNA signals in LUSC and normal samples at *BDH1* (F) and *PTPRZ1* (G) promoter and enhancer loci. The solid blue line at the bottom indicates the identified SOE.

Tumor tissue usually encompasses a mixture of multiple cells types, thereby averaging and diluting the transcriptional signals from the cancerous cells. Accumulative evidence had suggested that infiltrated stromal and immune cells influenced the genomic analysis of clinical tumor samples.^[^ ^32^, ^33^ ^]^ To better illustrate the transcriptional regulatory network in LUSC cells and identify key tumorigenic genes, we performed scATAC‐seq on six frozen LUSC tumor samples. Only the sequencing libraries passing the quality control were combined for subsequent analyses (detail see Methods). Cells can be clustered to 7 different cell types based on the similarity of ATAC peaks (Table S1D, Supporting Information). The cell type was annotated according to the chromatin‐accessibility in the promoters of marker genes (Figure S3, Supporting Information). In line with the previous reports, immune cells constituted a substantial portion of the tumor tissue composition, emphasizing the need for single‐cell analysis (**Figure** **2**A).^[^ ^34^, ^35^ ^]^ Notably, a cluster of cells showed strong expression of LUSC marker genes such as *TP63* and *SOX2*, which we annotated as squamous cells (Figure 2A). Interestingly, virtually all SOEs that we identified from the bulk tumor analysis displayed markedly high accessibility exclusively in the group of these squamous cells (Figure 2B), supporting that these squamous cells were primarily cancer cells. Furthermore, binding sites of SOX2, p63, as well as KLF5 and GRHL2 had all been enriched in the squamous cell cluster, further underscoring the importance of them as core TFs in mediating LUSC tumorigenesis (Figure 2C). Indeed, all of these TFs displayed squamous cell cluster‐specific expression pattern, as revealed by our integration of two external independent single‐cell transcriptomic datasets of LUSC (Figure S4, Supporting Information).^[^ ^34^, ^36^ ^]^ To examine the essential role of these four TFs particularly the two novel ones KLF5 and GRHL2, we depleted individual factors with siRNA in a human LUSC cell line HCC95 (Figure S5A–D, Supporting Information). The results consistently showed that cells deficient of any of these 4 factors except SOX2 were revealed with most prominent deficiency in epidermal cell development, an important feature of LUSC development (Figure S5E–H, Supporting Information). Note that SOX2 is also known to play a primary role in neuron development, and the depletion indeed caused disturbance in regulation of nervous system development.^[^ ^37^ ^]^ The pathways involved in epidermal cell development involving regulating keratinization and epithelial‐mesenchymal transition influencing tumor behavior, aggressiveness, and response to treatment.

In order to find out key oncogenes driven by SOEs, integrative analysis was performed using datasets from both bulk tissues and single cells. By intersecting the list of 3447 SOEs with 1945 squamous cell cluster specific ATAC peaks, we obtained 382 genomic fragments that were highly likely LUSC‐specific chromatin accessible loci. It turned out that 3036 genes were located within 200 kb of these loci, of which 122 genes exhibited specific upregulation both in bulk tumor and squamous cell cluster compared to adjacent normal tissues as well as other cell clusters within the tumor tissues, respectively (Figure 2D; Table S1E–G, Supporting Information). Given the important role of the four master TFs SOX2/p63/KLF5/GRHL2 revealed in binding and regulating the SOE activity, we reasoned that the SOE‐targeted oncogenes should undergo regulation of these TFs and thus exhibit a concerted oscillation of expression pattern with them. Among the 122 candidates, only 3 genes showed co‐expression pattern with all of the four master TFs (Figure 2E; Table S1H, Supporting Information), one of which was *NFE2L2* (Nuclear Factor Erythroid 2‐Related Factor 2, also known as *NRF2*), another well‐established oncogene in LUSC.^[^ ^38^ ^]^ In addition, we identified two novel candidates: D‐beta‐hydroxybutyrate dehydrogenase (*BDH1*; Figure 2F), a mitochondrial enzyme regulating the ketone body homeostasis during fatty acid catabolism and *PTPRZ1* (Figure 2G), a transmembrane enzyme specifically expressed in oligodendrocyte precursor cells.^[^ ^39^ ^]^ *BDH1* is located in the chromosome 3q29 region, which is highly amplified in the lung squamous cell carcinoma genome, suggesting that changes in *BDH1* expression are likely due to chromatin amplification (≈30% according to cBioPortal). In contrast, *PTPRZ1* was not detected for significant copy number amplification, implying that its elevated expression was likely driven by other mechanism, such as epigenetic remodeling. Therefore, we focused primarily on PTPRZ1 to investigate its therapeutic potential in treating LUSC.

### 2.3 SOEs Drive PTPRZ1 Expression in LUSC Cells

**Figure 3:**
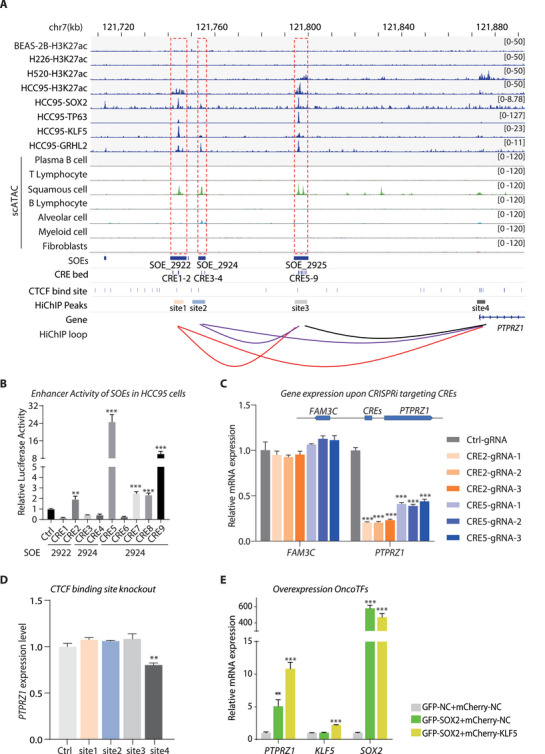
Regulation of PTPRZ1 by SOEs in LUSC. A) Genomic landscape of the *PTPRZ1* locus. Top: H3K27ac ChIP‐seq profiles in LUSC cell lines (BAES‐2B, H226, H520, HCC95). Middle: Binding patterns of transcription factors (SOX2, p63, KLF5, GRHL2) in HCC95 cells and scATAC‐seq from primary LUSC tissues at three SOEs adjacent to *PTPRZ1*. Bottom: Chromatin features including squamous oncogenic gained enhancers (SOEs_2922/2924/2925), *cis*‐regulatory element (CRE) clusters (CRE1‐9), CTCF binding sites, and chromatin interaction loops (HiChIP). B) Functional validation of enhancer activity at SOEs. Luciferase reporter assays in HCC95 cells show significant enhancer activity at SOE_2922 and SOE_2925. Relative luminescence was measured 48 hours post‐transfection (Firefly/Renilla ratio, normalized to empty vector control) (Data are shown as the mean ± s.e.m., n = 4 biological replicates; *P* values were assessed using two‐tailed Student's t tests; **P<0.01, ***P<0.001). C) PTPRZ1 expression in HCC95 cells was assessed by RT‐PCR following CRISPRi targeting of SOE_2922 and SOE_2925. gRNAs were designed against CRE2 in SOE_2922 and CRE5 in SOE_2925. Three highest‐scoring gRNAs per CRE were selected for CRISPRi. FAM3C, located upstream of these CREs, served as the control. (normalized to ACTB; Data are shown as the mean ± s.e.m., n = 4; *P* values were assessed using two‐tailed Student's *t*‐tests; **P<0.01, ***P<0.001). D) *PTPRZ1* transcriptional regulation via long‐range interactions between enhancers and its promoter. RT‐qPCR analysis demonstrates PTPRZ1 mRNA levels following CTCF binding site removal in H520 cells (Data are shown as the mean ± s.e.m., n = 3, *P* values were assessed using two‐tailed Student's t tests, **P<0.01). E) Induction of *PTPRZ1* expression by oncogenic TF cooperation. Relative mRNA expression levels of *PTPRZ1*, *KLF5*, and *SOX2* in BEAS‐2B cells following overexpression of oncogenic transcription factor SOX2 (GFP‐SOX2) alone and combined overexpression of SOX2 and KLF5 (GFP‐SOX2 + mCherry‐KLF5) compared to empty vector controls (normalized to ACTB; data are shown as the mean ± s.e.m., n = 3, *P* values were assessed using two‐tailed Student's t tests, **P<0.01, ***P<0.001).

Previous studies have shown that targeting super‐enhancer (SE)‐linked oncogenes could lead to significant tumor regression with potential applications in cancer therapy.^[^ ^40^ ^]^ In line, we found that there were three SOEs adjacent to *PTPRZ1* in the gene desert region, containing a total of nine *cis*‐regulatory elements (CREs), which were reported by the Encyclopedia of DNA Elements (ENCODE) Project.^[^ ^41^ ^]^ These SOEs were highly enriched for both H3K27ac signals and binding of the 4 abovementioned master TFs (**Figure** **3**A). Consistently, HCC95 and H520, two commonly studied LUSC cell lines, exhibited enriched H3K27ac signals at the *PTPRZ1* locus, resembling those found in LUSC patient tissues (Figure S6, Supporting Information). Thereby, we performed subsequent functional investigation in these two representative cell lines. First, we cloned and validated the function of individual CREs and confirmed that CRE2 in SOE_2922 and CRE5, 7, 8, 9 in SOE_2925 conferred strong *cis*‐regulatory activity in both HCC95 and H520 LUSC cell lines, indicating the primary role of these two SOEs in driving PTPRZ1 expression (Figure 3B; Figure S7A, Supporting Information). In comparison, SOE_2924 showed H3K27ac enrichment only in some of the LUSC tumor tissues but not in any of the LUSC cell lines examined (Figure S6, Supporting Information). In concert, the genomic fragment of SOE_2924 cloned to the reporter plasmid resulted in undetectable enhancer activity in both HCC95 and H520 cells (Figure S7A, Supporting Information). Next, we employed CRISPR interference (CRISPRi) to further verify the regulatory activity of these CREs, revealing differential levels of suppression of *PTPRZ1* expression among CREs, with the maximal downregulation observed upon CRE2 and CRE5 targeting.^[^ ^42^ ^]^ The results strongly supported the transcriptional activation of *PTPRZ1* by the enhancers within SOE_2922 and SOE_2925 (Figure 3C; Figure S7B, Supporting Information).

It is well established that the distal enhancer plays the regulatory role through long‐range chromatin interactions mediated by CTCF, which is essential for the stable expression of targeted genes.^[^ ^43^ ^]^ Indeed, all these SOEs contained multiple CTCF binding sites, and H3K27ac‐HiChIP result conducted in LUSC cells revealed significant chromatin interaction between PTPRZ1 promoter with all three adjacent SOE (Figure 3A).^[^ ^44^ ^]^ To further confirm the regulatory relationship, we leveraged CRISPR/Cas9 to transiently disrupt CTCF binding sites in HiChIP peaks of the three different SOEs in H520 cells (Figure S8, Supporting Information). Surprisingly, none of the individual deletion caused dramatic change of *PTPRZ1* expression except for the one located next to the *PTPRZ1* TSS (Figure 3D). To further examine the oncogenic role of the SOE‐associated master TFs in driving the expression of *PTPRZ1*, we simultaneously over‐expressed SOX2 and KLF5 in BEAS‐2B, an epithelial cell line that was isolated from noncancerous human bronchial epithelium. Note that the wildtype BEAS‐2B cells did not show signals of active enhancer marker H3K27ac in these CRE loci. The two TFs were previously reported to cooperatively establish a stably accessible region of chromatin.^[^ ^20^ ^]^ Consequently, the expression of *PTPRZ1* was significantly boosted (Figure 3E). The results suggested that multiple SOE co‐regulated the transcription of *PTPRZ1*, which ensured its robust expression in cancer cells.

### 2.4 PTPRZ1 Promotes Proliferation and Migration of Tumor Cells

**Figure 4:**
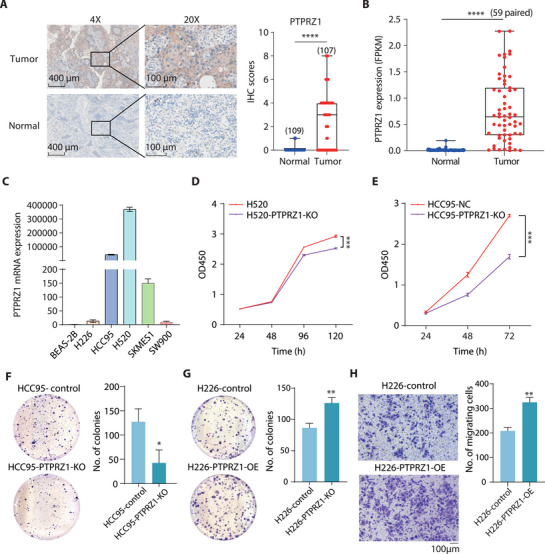
Role of PTPRZ1 in Tumor Cell Proliferation and Migration. A) Immunohistochemistry (IHC) analysis of PTPRZ1 protein expression in LUSC clinical specimens (Tumor, n = 109) compared to adjacent normal tissues (Normal, n = 107). Representative images are displayed (4×, scale bars: 400 µm; 20x, scale bars: 100 µm). Detailed IHC score calculation is provided in the methods section, with statistical analysis using the Mann‐Whitney test. B) Box‐scatter plot illustrating *PTPRZ1* expression levels from bulk RNA‐seq in tumor tissues versus adjacent normal tissues (59 pairs, student's t tests, ****P<0.0001). C) Comparative analysis of *PTPRZ1* expression in normal bronchial epithelial cells (BEAS‐2B) and five LUSC cell lines (H226, H520, HCC95, SKMES1, SW900), with data normalized to ACTB. D,E) CCK‐8 assay results showing the impact of PTPRZ1 on the proliferation of H520 and HCC95 control and knockout cell lines (Data are shown as the mean ± s.e.m., n = 6, *P* values were assessed using two‐tailed Student's *t*‐tests, ***P<0.001). F,G) Assessment of PTPRZ1's influence on clonogenic ability in HCC95/H226 control and HCC95‐KO/H226‐OE cell lines using a colony formation assay (Representative wells shown; quantification in right panels; data are shown as the mean ± s.e.m., n = 3, *P* values were assessed using two‐tailed Student's t tests, *P<0.05, **P<0.01). H) Evaluation of migration capacity in H226 cells via Transwell assay (Control versus PTPRZ1 overexpression). Migrated cells are stained with crystal violet (scale bars: 100 µm), n = 3, *P* values were assessed using two‐tailed Student's t tests, **P<0.01.

To investigate the tumorigenic role of PTPRZ1, we first examined the tumor biopsies and paired normal tissues using immunohistostaining, and observed a substantial elevation in the expression of PTPRZ1 at protein abundance, at a positive rate of 57.8% observed in tumor samples compared to merely 4.7% in normal tissues (**Figure** **4**A). Consistent results were also observed at the transcript level, including in both cancer tissues and various LUSC‐derived cell lines (Figure 4B,C; Figure S9, Supporting Information), but not detected in BEAS‐2B (Figure 4C). In addition, the single cell data also confirmed that *PTPRZ1* was specifically expressed in the tumor‐like squamous cell cluster, in a patten similar to the 4 master TFs (Figure S4B, Supporting Information). To investigate the potential role of the PTPRZ1 in tumor development, we leveraged CRISPR/Cas9 to perform knockout experiments in two distinct LUSC cell lines, H520 and HCC95, both characterized by high cellular expression of the gene (Figure S10A–D; Table S1I, Supporting Information). Following the disruption of PTPRZ1, we observed a significant reduction in the proliferation of both LUSC cell lines, as indicated by the Cell Counting Kit‐8 (CCK‐8) assay (Figure 4D,E). In alignment with the CCK‐8 results, the colony formation assay revealed that the capability of cells to form colonies was dramatically diminished after PTPRZ1 knockout (KO), further substantiating the role of PTPRZ1 in cell proliferation (Figure 4F). To reinforce our findings, we performed a colony formation assay in cells with ectopic overexpression (OE) of PTPRZ1. The LUSC cell line H226 was selected to maximize the observed effects as it displayed low native expression of PTPRZ1 (Figure 4C). Consistent with the knockout experiments, H226 cells with enhanced expression of PTPRZ1 showed a significant increase in proliferation (Figure 4G; Figure S10E, Supporting Information). In addition to promoting cell division, we also found that PTPRZ1 overexpression enhanced cell migration, demonstrated in the Transwell assay (Figure 4H), further indicating the oncogenic potential of PTPRZ1.

### 2.5 PTPRZ1 is Essential for Tumorigenesis In Vivo

**Figure 5:**
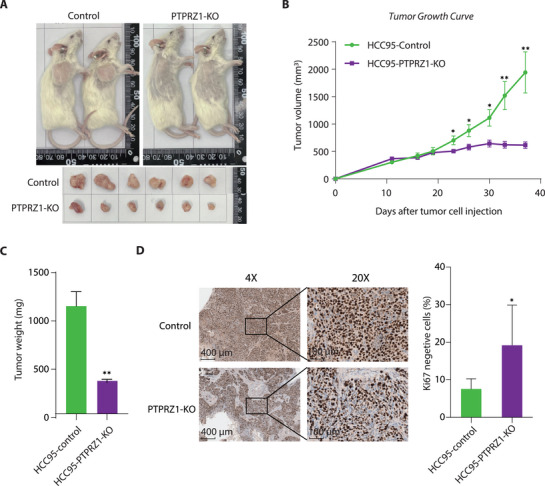
Critical Role of PTPRZ1 in LUSC Tumor Development in vivo. A) Photograph of tumor xenografts at endpoint, comparing control tumors to PTPRZ1‐KO tumors. Subcutaneous xenografts were established by injecting 1 × 10^8^ HCC95 control (empty vector) or PTPRZ1‐KO cells into the right flank of 6–8 weeks old female NCG mice (n = 6 per group). B,C) Tumor volume curves (B) and final tumor weights (C) for control versus PTPRZ1‐KO groups. Tumor growth was assessed via calliper measurements twice per week over 37 days, with volume calculated using the formula (length × width^2^)/2. The PTPRZ1‐KO group exhibited notably reduced tumor growth and final tumor mass compared to controls. Data are shown as the mean ± s.e.m., n = 6, *P* values were assessed using two‐tailed Student's t tests, *P<0.05, **P<0.01. D) Immunostaining of Ki‐67 in PTPRZ1 control and knockout tumor in the Xenograft model. Data are shown as the mean ± s.e.m., n = 6, *P* values were assessed using two‐tailed Student's t tests, *P<0.05.

Given the pivotal role of PTPRZ1 in driving cellular proliferation and motility in our cell model, we sought to interrogate its tumorigenic potential in vivo—a critical step toward validating its candidacy as a diagnostic and therapeutic target in LUSC. Therefore, we subcutaneously implanted PTPRZ1‐KO (knockout) and control HCC95 cells into the immunocompromised mice (n = 6). Strikingly, genetic ablation of PTPRZ1 unleashed a profound suppression of tumor growth, with KO tumors exhibiting a dramatic ≈70% reduction in volume compared to controls by day 37 post‐implantation (**Figure** **5**A,B, *P* < 0.01). This attenuation was further corroborated by a near threefold decrease in tumor weight at endpoint (Figure 5C, *P* < 0.01), underscoring PTPRZ1's indispensable role in sustaining tumor mass. We also carried out the experiments using H520 cells with PTPRZ1‐KO and control, and the trend of reduced tumor growth was consistently observed in the PTPRZ1‐disrupted cells (Figure S11, Supporting Information). The attenuated knockout phenotype in H520 cells compared to HCC95 cells may be attributable to the closer resemblance of epigenetic modifications at the *PTPRZ1* enhancer in HCC95 than H520 cells to those in tumor tissues (Figure S6, Supporting Information). Furthermore, H520 cells are originated from a primary lung tumor, whereas HCC95 cells are derived from metastatic tumor. This suggests that HCC95 cells likely exhibit a more aggressive phenotype than H520 cells, characterized by higher proliferative and migratory capacities. Notably, this phenotype was consistent across all mice, with no outliers, signifying the robustness of PTPRZ1 as a driver of LUSC progression. Immunohistochemical analysis of PTPRZ1 KO tumors revealed a stark decline in proliferative activity, as evidenced by diminished Ki‐67 staining (Figure 5D). Collectively, these compelling in vivo findings position PTPRZ1 as a key player of LUSC malignancy—orchestrating proliferation, survival, and metastatic competency. Our data not only solidify its candidacy as a prognostic biomarker but also ignite promise for therapeutic strategies targeting PTPRZ1 to cripple tumor growth and improve clinical outcomes in LUSC patients.

### 2.6 MDK is the Potential Ligand of PTPRZ1 in LUSC Tumorigenesis

PTPRZ1 is a cell surface receptor phosphatase that would be inactivated by binding to an extracellular ligand, which consequently led to sustained phosphorylation of its downstream cellular substrate. There had been a few known ligands for PTPRZ1 including pleiotrophin (PTN), midkine (MDK), neuronal cell adhesion molecule (NRCAM), interleukin 34 (IL34), Contactin 1 (CNTN1), NCAN, PGCB.^[^ ^45^, ^46^ ^]^ For example, in glioblastoma multiforme (GBM), tumor‐associated macrophage (TAM) secreting PTN binds to PTPRZ1 and thus maintains sustained tyrosine phosphorylation of downstream substrates, including β‐catenin and receptor tyrosine kinases (RTKs), thereby activating oncogenic pathways.^[^ ^47^ ^]^ By disrupting the interaction between PTPRZ1 and its inhibitory ligand would prevent cancer cell proliferation. Therefore, finding the key ligand and evaluate whether its binding to PTPRZ1 was essential for the tumor growth of LUSC could provide potential therapeutic target to treating the disease.

**Figure 6:**
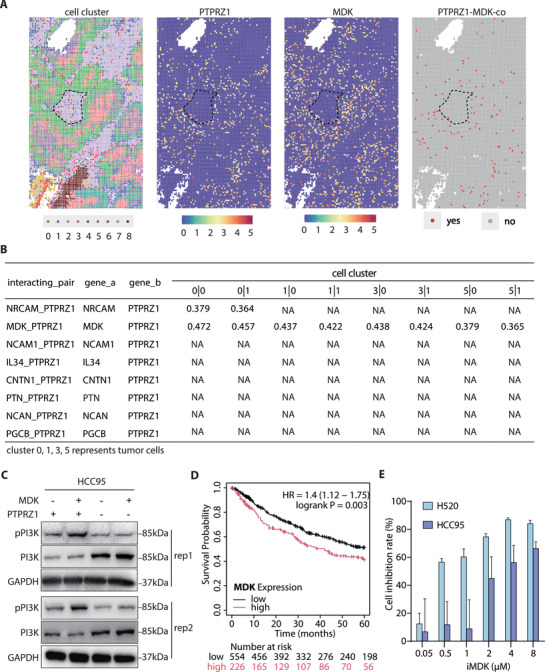
MDK as a Potential Ligand for PTPRZ1 in LUSC Tumorigenesis. A) Spatial transcriptomics analysis indicating co‐localization of *PTPRZ1* and *MDK* expression in tumor regions using 10x Genomics Visium HD. Single‐cell expression profiles show PTPRZ1 and its co‐receptor MDK distributed across tumor microenvironment clusters (0‐8), with co‐expressing cells marked in red and non‐co‐expressing cells in gray. B) Quantitative analysis of ligand‐receptor interactions using CellPhoneDB v5.0 across 9 cell clusters from spatial transcriptomic data. The table presents mean expression values of significant gene pair interactions in tumor cell clusters (0, 1, 3, and 5), with mean expression values only shown for significant interactions (P < 0.05). Non‐significant interactions are marked as “N/A”. Results identify MDK‐PTPRZ1 as the top significant interacting pair among tested candidates. C) Immunoblot analyses of phospho‐PI3K and total PI3K expression in control versus *PTPRZ1*‐KO HCC95 cells following recombinant MDK stimulation. GAPDH serves as a loading control for normalization. Data from two biological replicates are displayed. D) Clinical relevance of MDK signaling assessed through Kaplan‐Meier analysis, revealing worse overall survival in LUSC patients with high MDK expression levels (HR = 1.4; log‐rank P = 0.003). E) Pharmacological inhibition of MDK illustrated by dose‐response histograms showing significant growth suppression in H520 and HCC95 cells treated with the MDK inhibitor (iMDK, HY‐110171) for 72 hours, as assessed by CCK‐8 assay (mean ± s.e.m., n = 6).

The single cell analysis has revealed high heterogeneity of LUSC tumor tissue and the tumor‐specific expression of the 4 master TFs as well as their target gene *PTPRZ1*. However, it remained unclear the spatial distribution of cancer cells within the tumor and how the cancer cells were interacting with other cell types in the tumor microenvironment. To find out the primary oncogenic ligand of PTPRZ1 in LUSC, we performed spatial transcriptomic analysis of LUSC using 10x Genomics Visium HD system. According to the transcriptomic profiles, we were able to isolate 9 different cell clusters (**Figure** **6**A left 1, Figure S12, Supporting Information). Clusters 0, 1, 3, 5 and 6 were dominated by the tumor‐like squamous cells, which were characterized by squamous cell marker gene expression (Figure S12A, Supporting Information) and cluster‐specific differentially expressed genes GO enrichment analysis (Figure S13A, Supporting Information). Note that *PTPRZ1* was highly and specifically expressed in these tumor cells (Figure 6A, left 2). Interestingly, tumor cells in the front edge facing these immune cells (cluster 0) exhibited significant activation of mitotic division, suggesting that they were rapidly dividing cells. A large number of immune cells, designated as cluster 2, were present in the tumor tissue surrounding the tumor cells, consistent with the single‐cell result (Figure S13B, Supporting Information). The cluster 6 contained cells showing activation of ameboidal−type cell migration pathway, and were mostly interspersed from clouds of cancer cells, indicating these were likely invasive tumor cells.

To this end, the information of high‐resolution spatial transcription of genes allowed us to fine‐map the ligand‐receptor of PTPRZ1 in LUSC. We analyzed the interaction specificity between PTPRZ1 and its potential ligands based on their mean expression in the tested cell cluster pairs extracted from the spatial transcriptome data using CellPhoneDB v5.0. Mean expression values of significant gene pair interactions (*P* < 0.05) in tumor cell clusters (0, 1, 3, and 5) are presented in Figure 6B, while clusters failing to meet this significance threshold (*P* ≥ 0.05) were excluded. The result showed that MDK was the most significant candidate ligand interacting with PTPRZ1 among tumor cells, consistent with their co‐expression patterns (Figure 6A; Figure S4B, Supporting Information). Previous research has demonstrated that the interaction between MDK and PTPRZ1 promotes epithelial‐mesenchymal transition (EMT), characterized by a decrease in E‐cadherin and an increase in Vimentin.

To unravel the downstream signalling pathway activated by MDK‐PTPRZ1, we exogenously added recombinant MDK and revealed a significant increase in PI3K phosphorylation, an effect abolished upon PTPRZ1 knockout (Figure 6C). These results demonstrated that MDK promotes PI3K phosphorylation via PTPRZ1, thereby driving cell proliferation. These findings also indicate that targeting the MDK‐PTPRZ1 axis—for example through methods such as monoclonal antibodies, small‐molecule inhibitors, or ligand traps—could disrupt oncogenic signaling and make tumors more responsive to chemotherapy. Indeed, high levels of MDK are associated with poor survival rates in LUSC (Figure 6D). And, when H520 and HCC95 cells were treated with MDK inhibitors, their proliferation was significantly reduced (Figure 6E), underscoring the therapeutic potential of this approach in treating LUSC.

## 3 Discussion

This study delineates a comprehensive landscape of 3447 lung squamous cell carcinoma (LUSC)‐specific oncogenic enhancers (SOEs), unveiling their pivotal role in driving tumor pathogenesis through enrichment of key transcription factors, including SOX2, p63, KLF5, and GRHL2. These SOEs orchestrate transcriptional programs that fuel malignant progression. Leveraging an integrative multi‐omics framework—spanning ChIP‐seq, bulk RNA‐seq, single‐cell ATAC/RNA‐seq, and spatial transcriptomics—we identified *PTPRZ1* as a previously unrecognized SOE‐driven oncogene. PTPRZ1 exhibited robust tumor‐specific expression, with functional assays demonstrating its critical role in accelerating cellular proliferation and migration in vitro. Strikingly, *PTPRZ1* genetic ablation achieved potent tumor growth suppression (≈70%) in xenograft models, underscoring its therapeutic relevance in vivo. Spatial transcriptomic profiling further resolved the functional interplay between PTPRZ1 and its dominant ligand, midkine (MDK). Exogenous recombinant MDK significantly increased PI3K phosphorylation – an effect abolished by PTPRZ1 knockout – while MDK inhibition disrupted proliferative signaling cascades. These findings reveal the MDK‐PTPRZ1 axis as a linchpin of LUSC progression, which collectively illuminate the centrality of enhancer‐driven regulatory networks in LUSC biology while exemplifying the transformative potential of multi‐omics integration in unearthing novel biomarkers and targetable vulnerabilities. By bridging epigenomic dysregulation to actionable therapeutic strategies, this work advances precision oncology paradigms for LUSC, a malignancy historically plagued by limited clinical interventions.

LUSC, one of the leading causes of cancer‐related mortality worldwide, is characterized by several well‐defined genetic driver mutations. However, in contrast to malignancies such as LUAD, in which targeted therapy approaches have been encouraging, translating similar strategies for LUSC has largely failed in the clinics.^[^ ^6^ ^]^ The biomarker‐driven clinical trials for LUSC evaluated in the Lung Cancer Master Protocol (Lung‐MAP; S1400) failed to show an improvement of therapeutic effect of currently available targeted therapy with an overall response rate of only 7%.^[^ ^48^ ^]^ Furthermore, inhibitors against emerging signaling pathways in LUSC such as PI3K pathway, CDK4/6 pathway and FGFR1 driver mutations, have also led to poor clinical benefits.^[^ ^38^ ^]^ Recently, immune checkpoint blockades via antibodies that block the inhibitory immune‐checkpoint proteins, such as programmed cell death protein 1 (PD‐1) or its ligand (PD‐L1), have emerged as a key component of the standard of care treatment for LUSC, but the overall response rate remains low.^[^ ^49^, ^50^ ^]^ Therefore, it is of vital importance to have a better understanding of the mechanisms underlying the pathogenesis of LUSC as well as identify effective therapeutic approaches. Genetic changes as a therapeutic target for squamous cell lung carcinoma have reached a bottleneck, and epigenetic changes may be a promising direction. Previous epigenetic studies in lung squamous cell carcinoma (LUSC) have primarily examined the impact of dysregulated epigenetic regulators on tumor progression through analyses of global epigenetic modifications.^[^ ^51^, ^52^ ^]^ While Jiang et al. showed that TP63 and SOX2 drive oral squamous cancer progression via super‐enhancers (consistent with our findings), their work utilized cell line models.^[^ ^19^ ^]^ Unlike these studies, this work represents the first large‐scale, whole‐genome epigenomic profiling of patient‐derived tissue samples to identify novel driver genes in LUSC.

Substantial evidence demonstrates that transcription factors play critical roles in the functional activity of enhancers; our integrative analysis of patient‐derived samples extends this understanding to clinical contexts. Recent findings reveal that conserved regulatory networks exist across various squamous cell carcinomas (SCCs). Notably, LUSC shares essential transcriptional regulators—including SOX2, p63, KLF5, and GRHL2—with other SCCs, such as esophageal squamous cell carcinoma (ESCC) and head and neck squamous cell carcinoma (HNSCC).^[^ ^19^ ^]^ These transcription factors play a pivotal role in orchestrating enhancer‐driven oncogenic programs, thereby modulating critical signaling pathways, including NFκB and the epithelial‐mesenchymal transition, both of which are vital in the pathogenesis of SCCs. This molecular convergence positions the MDK‐PTPRZ1 axis as a promising therapeutic target across multiple SCC subtypes. Although several PTPRZ1 inhibitors exist (e.g., NAZ2329, MY33‐3), our unpublished data showed they lacked efficacy in cell derived xenograft (CDX) models, potentially due to challenges in targeting this membrane receptor. Given MDK's essential function in normal tissue development, strategies aimed at disrupting the MDK‐PTPRZ1 interaction appear highly promising. Approaches such as high‐throughput screening of small‐molecule inhibitors or the development of neutralizing antibodies against this interaction represent particularly viable research directions. For instance, Zhang et al. demonstrated that curcumin could effectively block the binding of pleiotrophin (PTN) to PTPRZ1, thereby inhibiting neuroinflammation post‐epileptic seizures.^[^ ^53^ ^]^ Building on this knowledge, our study proposes that the development of small chemical compounds or monoclonal antibodies aimed at inhibiting the MDK‐PTPRZ1 interaction may provide an effective strategy to attenuate the progression of LUSC and potentially other SCCs, offering the prospect of broad therapeutic efficacy. Furthermore, the significant association of CDEs with “hemopoiesis, cytokine production and lymphocyte activation, *etc*” strongly indicated that the epigenetic reprogramming‐mediated reduced immunity was another crucial factor in driving LUSC. Therefore, it is believed that combining interference against MDK‐PTPRZ1 interaction with immunotherapy holds promise for achieving superior therapeutic outcomes. In summary, our work illustrates enhancer addiction as a hallmark of LUSC, identifies PTPRZ1‐MDK as a pivotal epigenetic modulator axis for oncogenesis, and establishes a paradigm for dissecting epigenetic vulnerabilities in solid tumors through multi‐omics integration. Our work provides an an effective strategy in inhibiting the MDK‐PTPRZ1 interaction in LUSC in future clinical utility.

## 4 Experimental Section

### Patient Sample Collection and Clinical Data

The discovery cohort has recruited 109 patients with LUSC at the Cancer Hospital, Chinese Academy of Medical Sciences, from December 2017 to December 2019. Each freshly frozen tumor specimen had a matched adjacent normal sample that was obtained from at least 2 cm away from the tumor. The enrolled patients received no prior treatment before surgical resection. Histologic sections from tumor and adjacent normal tissues were obtained for review. This study was approved by the institutional review board of Cancer Hospital, Chinese Academy of Medical Science (Approval Number: NCC20240‐045). Informed consent was obtained from all patients.

### Mice Samples

NCG mice, which are triple‐immunodeficient, lack functional/mature T, B, and NK cells, and have reduced macrophage and dendritic cell function, were purchased from GemPharmatech Co., Ltd, China, and were used for xenograft experiments. NCG mice were raised under strict pathogen free (SPF) conditions in a barrier facility, with temperature (22 – 26 °C), humidity (40 – 70%), HEPA filtration positive pressure ventilation, and 12 h of light/dark cycle to maintain mice health and experimental reproducibility. The xenograft experiments were conducted following approval by the Institutional Animal Care and Use Committee (IACUC) of innomodels Biotechnology (Beijing) Co., Ltd., with the approval number IACUC‐003‐2024‐0012.

### Cell Lines and Culture

Human squamous cancer cell lines include H520 (ATCC catalog No. HTB‐182), HCC95 (Merck catalog No. SCC483), H226 (ATCC catalog No. CRL‐5826), SKMES1(ATCC catalog No. HTB‐58), SW900 (ATCC catalog No. HTB‐59), along with human normal lung epithelial cells BEAS‐2B (ATCC catalog No. CRL‐9609) and human renal epithelial cells HEK293T (ATCC catalog No. CRL‐3216). H520‐KO‐PTPRZ1 cell were all purchased from Hysigen Bioscience (Catalog No. CGKO‐M2278). All cells were cultured in a medium supplemented with 10% fetal bovine serum, with specific mediums used for different cell lines. DMEM (Gibco, C1199550BT) was used for BEAS‐2B, SW900 and HEK293T cell lines, RPMI 1640 (Gibco, C11875500BT) was used for H520, H520‐KO‐PTPRZ1, HCC95 and H226 cell lines, and MEM (Gibco, C12571500BT) was used for SKMES1. All cells were maintained in a cell culture incubator at 37 °C with 5% CO_2_.

### ChIP Assay and ChIP‐Sequencing

ChIP assays were carried out as previously described.^[^ ^54^ ^]^ Briefly, around sixty milligrams of each tissue were cut into 1 mm^3^ pieces in PBS with protease inhibitor. Tissue pieces or cancer cells were fixed with 1% formaldehyde (F8775, Sigma) for 10 min at room temperature, and a final concentration of 125 mm glycine was used to quench the reaction. Cells were washed twice with cold PBS and collected in cold PBS by scraping, then resuspended in hypotonic lysis buffer (20 mm Hepes, pH 7.9, with 10 mm KCl, 10% glycerol, 1 mM DTT and a protease inhibitor cocktail (04693124001, Roche)). The pellet of nuclei was resuspended in RIPA buffer (10 mm Tris‐HCl, pH 8.0, with 140 mm NaCl, 1% Triton X‐100, 1 mM EDTA, 0.1% SDS, 0.1% sodium deoxycholate and Protease Inhibitor). Nuclear extracts were sonicated to generate chromatin fragment on a Covaris M220 Focused‐ultrasonicator (Insert, microTUBE 130 µL; Temperature, 7 °C; Peak Incident Power, 75W; Duty Factor, 5%; Treatment Time, 300s). The chromatin was cleaned by centrifugation at 14000 × g for 10 min at 4 °C and diluted with 1 mL RIPA buffer per reaction. Then the chromatin was precleared with Protein G Sepharose 4 Fast Flow (17061801, GE) for 2 h, 30 µL of sample was set aside as an input and subjected to immunoprecipitation with antibody against H3K4me1 (ab8859, Abcam), H3K27ac (T59439M, Abmart), TP63 (13109S, Cell Signaling Technology), SOX2 (2748S, Cell Signaling Technology), KLF5 (ab137676, Abcam), GRHL2 (ab271023, Abcam) overnight. On the following day, Protein G Sepharose 4 Fast Flow that were blocked with RIPA containing 0.5% bovine serum albumin (BSA) were added into the samples followed by incubation for 2 h in cold room under rocking. The beads were then washed three times with RIPA buffer, followed by two times in RIPA buffer with 0.5 m NaCl, once in LiCl buffer [250 mm LiCl, 1 mM EDTA, 0.5% IGEPAL CA‐630,0.1% sodium deoxycholate, and 10 mM tris‐HCl (pH 8.0)], and twice in ice‐cold Tris‐ EDTA (TE) buffer. Each time, the beads were sustaining for 5 min with a gentle rock. After washing, both the beads and input sample were added with 150 µL of extraction buffer (1% SDS in 1× TE, 12 µL of 5 M NaCl, and 10 µg of RNAse A) and incubated at 37 °C for 1 h with a shaking. Next, 20 µg of Proteinase K was added to the samples for incubation at 65 °C overnight with thermoshaking. DNA was isolated by performing a phenol/chloroform/isoamyl alcohol extraction to prepare the ChIP‐seq library using the NEBNext Ultra II DNA Library Prep Kit (NEB, E7645). The library was sequenced using the DNBSEQ‐T7 PE150 platform (Biopharmaceutical PublicService Platform, Nanjing).

### ChIP‐seq Data Processing

The ChIP‐seq reads were mapped into the human reference genome (hg38) using Bowtie2 (version 2.5.0). Matching input control was used to call peaks. Peak calling was performed using MACS3. For tissues and cells H3K27ac and H3K4me1 data, a “–broad –broad‐cutoff 0.1” was used for peak calling; For TP63, SOX2, GRHL2, KLF5 ChIP‐seq data, a “‐q 0.01” was used for peak calling. Bigwig tracks were generated using deepTools (version 3.5.1) with (Reads Per Kilobase of transcript per Million mapped reads (RPKM) normalization and visualization with Integrative Genomics Viewer (IGV;  version 2.12.3).

### Enhancer Identification

For both H3K4me1 and H3K27ac ChIP‐seq data, it first identified the common peak of H3K4me1 and H3K27ac, then the peaks away from the ± 2.5 kb flank region of transcriptional start sites (TSS) were identified as enhancers. At the same time, enhancers that could not be identified in at least two samples were excluded from further analysis. Then the enhancers of each sample were merged into one single set and featureCounts (version 2.0.1) was used to count at enhancer region in each sample. We calculated the normalized RPKM as the enhancer signal in a specific region. Differential enhancer analysis between normal and tumor tissue was performed using paired t test. Enhancers whose log_2_(Tumor/Normal) > 0.75 and padj < 0.05 were identified as LUSC onco‐enhancers (SOEs), log_2_(Tumor/Normal) < −0.75 and adjusted *P* < 0.05 were identified as cancer‐depleted enhancers (CDEs).

### Identification and GO Analysis of Genes Associated with Enhancer

The coordinate file of SOEs and CDEs were submitted to the Genomic Regions Enrichment of Annotations Tool (GREAT) website (version 4.0.4) and the software will consider the nearest gene as regulatory gene to perform biological process (GO) analysis. URL to GREAT analysis: https://great.stanford.edu/great/public/html/

### Prediction of Enriched TFs on Gain Enhancer

The common region of SOEs and LUSC ATAC‐seq peak was defined as gain enhancer nucleosome‐free regions (NRFs), and the common region of CDEs and normal human ATAC‐seq peak (https://www.encodeproject.org/files/ENCFF818EYD/) as loss enhancer NRFs.^[^ ^55^ ^]^ HOMER (v 4.1.1) software plugin findMotifsGenome.pl was used to calculate the significance of TFs enrichment on nucleosome‐free regions (NRFs) of SOEs, which use CDEs NRFs as background.

### scATAC‐seq Library Preparation

ScATAC‐Seq was performed on six frozen LUSC tissues by NovogeneCo., Ltd. (China). For scATAC‐seq, fresh nuclei were isolated with 1 mL prepared lysis buffer (10 nm Tris‐HCI, 10 mm NaCI, 3 mm MgCl2, 0.1% NonidetTM P40 Substitute (#74385; Sigma–Aldrich)) and incubated on ice for 5 min. The culture was centrifuged at 500× g for 5 min at 4 °C and mixed with 1 mL nuclear resuspension buffer (1× PBS, 1% BSA, and 0.2 U µL^−1^ RNase Inhibitor). The homogenate was filtered through a 40‐µm cell strainer (#H13680‐0040; BelArt) and the nuclear concentration was determined using Countess II FL Automated Cell Counter (#C10228; Thermo Fisher). Nuclei were then immediately used to generate single cell ATAC‐seq libraries.10× Chromium libraries were prepared according to manufacturer protocol. Six scATAC‐seq libraries were prepared according to the Chromium NextGEM Single Cell ATAC Reagent Kits v1.1 User Guide RevC (10x Genomics CG000209). All libraries were sequenced on Illumina NovaSeq 6000 using a paired‐end 150 bp protocol.^[^ ^56^ ^]^

### scATAC‐seq Data Analysis

Raw sequencing data were processed with Cell Ranger ATAC (v2.1.0, 10x Genomics) software for demultiplexing, aligning to GRCh38 human reference genome, and generating peak‐barcode matrices. Signac (v1.10.0) R package was used to perform subsequent analysis. Retain high‐quality cells according to the following criteria: peak widths > 20, peak widths < 10000, peak region fragments > 3000, peak region fragments < 20000, reads in peaks > 15%, blacklist ratio < 0.05, nucleosome signal < 4 and TSS enrichment > 3. After filtering, a dataset from two samples comprising 148828 peaks from 19556 cells remained for latent semantic indexing (LSI) analysis.

The activity of each gene was quantified by examining the local chromatin accessibility, including the 2 kb upstream of the transcriptional start site and gene body, a scaling factor was computed for each sample, defined as the median number of transposition sites in promoters across cells, and scores were then log‐normalized and scaled such that the sum in each cell equals the scaling factor. Clustering and dimensionality reduction were then performed on the corrected LSI components by the Harmony method. One cluster (9) were excluded because they contained fewer than 100 cells. Finally, 9 clusters were obtained with resolution  =  0.5 and dims  =  2:15. To help to interpret the scATAC‐seq data, the gene activity matrix was obtained according to the above, in which gene coordinates are extracted and extended to include the 2 kb upstream region, and scored each cluster by the normalized expressions of the LUSC markers mentioned in scRNA‐seq analysis. Differential chromatin accessibility analysis was performed by the “FindAllMarkers” function. Differentially accessible chromatin regions were identified with Bonferroni‐adjusted *P* values smaller than 0.05 and log_2_‐fold‐change values larger than 0.25.

TF binding enrichment analysis was mainly based on the chromVAR (v 1.20.2) R package. In brief, a list of motif position frequency matrices was obtained from the JASPAR database, and it ran the *AddMotifs* function to add the DNA sequence motif information required for motif analyses. Then, this could calculate a per‐cell motif activity score by running chromVAR and identify differential activity scores between cell types. Motif activity scores were normalized by z‐scores, and the differential activity scores between cell types were replaced with “avg_diff.”.

### RNA‐seq Library Construction

Total RNA was isolated with TRIzol reagent (15596018, Invitrogen) from cultured cells. For RNA‐seq library preparation, poly (A)+ RNA was selected using the NEB‐Next Poly(A) mRNA Magnetic Isolation Module (NEB, E7490L) to perform the LM‐seq procedure.^[^ ^57^ ^]^ including that isolated mRNA is fragmented in reverse transcription buffer with heat and then reverse‐transcribed with SmartScribe reverse transcriptase (Clontech) using a random hexamer oligo (HZG883). After reverse transcription, RNA is removed by RNaseA and RNaseH treatment. A partial adaptor (HZG885) is then ligated to the single stranded cDNA using T4 RNA ligase 1 (NEB) and incubated overnight at 22 °C. After purification, ligated cDNA is amplified by 18 cycles of PCR using oligos that contain full BGI adaptors. Last, the resultant libraries were purified using the DNA Clean Beads, followed by sequencing using the DNBSEQ‐T7 PE150 platform (Biopharmaceutical PublicService Platform, Nanjing).

### RNA‐seq Data Processing and GO Analysis of DEG

The RNA‐seq reads were aligned to the human reference genome (hg38) using HISAT2 (2.2.1) with default settings. Uniquely aligned reads were counted at gene regions using the package featureCounts (version 2.0.1) based on Gencode v40 annotations.

For tissues RNA‐seq data, RPKM was used to normalize the gene expression matrix. Differential expressed genes (DEGs) analysis between paired normal and tumor tissue was performed using the Wilcoxon Signed‐Rank Test, and Wilcoxon rank sum test was used to identify DEGs between unpaired normal and tumor tissue. Genes whose log2(Tumor/Normal) > 1 and padj < 0.05 were identified as up genes, log2(Tumor/Normal) < −1 and adjusted *P* < 0.05 were identified as down genes. The GO analysis was performed by DAVID database (v2023q4).

For RNA‐seq data of TP63, SOX2, KLF5 and GRHL2 knocked down (KD) in HCC95 cell line, The R package DESeq2 (version 1.40.0) was used to identify DEGs between KD samples and NC samples. Genes whose abs(log2FC) > 0.75 and *P*_adj_ < 0.05 were identified as DEGs. The GO analysis was performed by clusterProfiler (v4.8.3).

### Single‐Cell RNA‐seq Analysis

LUSC scRNA‐seq data was obtained from two previous published studies.^[^ ^34^, ^36^ ^]^ Seurat (v4.0.3) R package was used to perform filtering, normalization, dimensionality reduction, clustering, and differential expression analysis. This further selected high‐quality cells to be preserved, with the following criteria: 1) gene number between 200 and 6000; 2) Unique molecular identifiers (UMI) count > 1000; 3) mitochondrial content < 25%. Doublets were predicted by the DoubletFinder (v2.0.4) algorithm. After filtering, a total of 261765 cells were left. The batch effect across different samples was eliminated by the Harmony (v1.1.0) method. The top 20 harmony embedding was selected by the “ElbowPlot” function and used to perform clustering and visualization. The “FindClusters” function was performed to generate different clustering results with resolutions ranging from 0.2 to 1.2. An appropriate resolution was determined based on cluster stability with clustree (v0.5.1) R package. Finally, 17 clusters (resolution  =  0.2) were obtained. One cluster (16) were excluded because they contained fewer than 100 cells or did not express known markers, and 261667 cells were retained for the subsequent analysis.

To assign one of the 10 major cell types to each cluster, each cluster was scored by the normalized expressions of the following canonical markers: B cells (*CD79A*, *CD79B*, *MS4A1*), Brush cells (*SPTSSB*, *ACADSB*, *COLCA2*, *HOMER3*, *NFATC3*), Ciliated airway epithelial cells (*TPPP3*), Myeloid cells (*CD68*, *LYZ*), Plasma B cells (*MZB1*, *JCHAIN*, *IGHG1*), T cells (*CD2*, *CD3D*, *CD3E*, *CD3G*), Fibroblasts (*COL1A1*, *COL1A2*, *DCN*), Alveolar cells (*CLDN18*, *AQP4*), Squamous cells (*SOX2*, *SERPINB5*), Mast cells (*GATA2*, *TPSAB1*, *TPSB2*), Mast cells (*GATA2*, *TPSAB1*, *TPSB2, TPSAB1, CPA3*). The highest scored cell type was assigned to each cluster. The clusters assigned to the same cell type were lumped together for the following analysis. The final results were manually examined to ensure the correctness of the results and visualized by Uniform Manifold Approximation and Projection (UMAP). The 10 major cell types were chosen by initial exploratory inspection of the differentially expressed genes (DEGs) of each cluster combined with literature study. Differential gene analysis was performed by the Seurat “FindMarkers” function. DEGs were identified with Bonferroni‐adjusted *P* values < 0.05 and log_2_‐fold‐change values > 0.25.

### 10× Genomics Visium HD Library Preparation and Sequencing

10x Genomics Visium HD was performed on frozen LUSC tissues by Novogene Co., Ltd. (China). Ten‐micrometre cryosections were cut and placed on Visium slides, then processed according to the manufacturer's instructions. In brief, sections were fixed with cold methanol, H&E stained and imaged on a Hamamatsu NanoZoomer S60 before permeabilization, reverse transcription and cDNA synthesis using a template‐switching protocol. Second‐strand cDNA was liberated from the slide and single‐indexed libraries were prepared using a 10x Genomics PCR‐based protocol. Library were sequenced on Illumina Hiseq using a paired‐end 150 bp protocol.

### 10× Genomics Visium HD Spatial Transcriptomic Analysis

Raw FASTQ files and histology images were processed by SpaceRanger (v3.1.1, 10x Genomics) on the reference GRCh38 human genome to estimate gene expression on spots. Seurat (v4.0.3) was used to perform filtering, normalization, visualization, unsupervised clustering, differential expression analysis. High‐quality cells were further selected to be preserved, with the following criteria: 1) gene number > 300; 2) UMI count > 300; 3) mitochondrial content < 25%. After filtering, a total of 100045 cells were left to perform normalization. Then a set of 50000 cells was sketched form the Visium HD dataset, perform clustering on the subsampled cells, and then project the cluster labels, and dimensional reductions (PCA and UMAP) that learned from the 50000 sketched cells – to the entire dataset, using the “ProjectData” function. Finally, 9 clusters (resolution  =  0.5) were obtained. Differential gene analysis was performed by the Seurat “FindMarkers” function and GO analysis was performed by clusterProfiler(v4.8.3). Tumor cell cluster was identified according to GO analysis results and average expression values of some cancer stem cell marker (*CD24*, *CD44*, *EPCAM*, *MUC1*, *SOX2*) in different clusters.

### Ligand–Receptor Analysis

The CellPhoneDB (v5.0.0) was used to investigate cellcell interaction between different cell types, especially in tumor cells in LUSC.^[^ ^58^ ^]^ To capture interaction more systematically and comprehensively, various ligand‐receptor resources were integrated in CellPhoneDB database, and added six ligands interacting with PTPRZ1 from STRING (Search Tool for the Retrieval of Interacting Genes/Proteins; v 12.0) database, and finally got 2924 human ligand‐receptor pairs. The gene‐cell raw matrix data, cell type annotation information, and these ligand–receptor pairs were input to CellPhoneDB with threshold  =  0.1. The significant ligand–receptor pairs with *P* < 0.05 were selected for subsequent analyses.

### Lentivirus Production and Cell Transduction

For lentiviral production, 293T cells were seeded in 10 cm dishes in complete DMEM medium (Gibco) 24 h prior to transfection. At ≈70% confluency, 293T cells were transfected with a plasmid mixture containing 4 µg transfer plasmid, 2 µg psPAX2 packaging plasmid (Addgene #12260), and 1 µg pMD2.G envelope plasmid (Addgene #12259) in 1 mL Opti‐MEM (Gibco) using 21 µg polyethylenimine (PEI, Polysciences). The DNA‐PEI complex was incubated at room temperature for 15 min before adding to cells. After 12–24 h, the medium was replaced with 10 mL fresh, pre‐warmed antibiotic‐free DMEM. Sodium butyrate (10 mM final concentration) was added 24 h post medium change to enhance viral production. Viral supernatants were collected at 48 h and 72 h post‐transfection, centrifuged at 800 × g for 5 min, and filtered through 0.45 µm PVDF filters (Millipore). The pooled supernatants (≈12 mL) were mixed with 3 mL PEG8000 (Sigma) and incubated at 4 °C overnight. Viruses were concentrated by centrifugation at 4000 × g for 20 min at 4 °C (slow acceleration/deceleration). The pellet was resuspended in 150 µL Opti‐MEM, aliquoted (50 µL), and stored at ‐80 °C.

For cell transduction, target cells were seeded in 6‐well plates in antibiotic‐free medium 24 h prior to infection. At ≈50% confluency, cells were incubated with viral supernatant (20‐40 µL) and 20 µL HitransG P (REVG005, GeneChem) in 0.5 mL Opti‐MEM for 8–12 h, followed by replacement with complete medium. Cells were split 1:2 at 48 h post‐transduction for selection or cryopreservation.

### CRISPRi‐Mediated Enhancer Silencing, CRISPR‐Cas9 Mediated Knockout, and Lentiviral‐Mediated Overexpression of Gene

pLV hU6‐sgRNA hUbC‐dCas9‐ZIM3‐KRAB‐P2A‐EGFP‐T2a‐Puro vector was used for CRISPR KD experiments. sgRNAs, which were designed for each locus with the https://chopchop.cbu.uib.no/ design tool, were cloned into pLV hU6‐sgRNA hUbC‐dCas9‐ZIM3‐KRAB‐P2A‐EGFP‐T2a‐Puro vector. LentiCRISPR v.2 vector was used for CRISPR KO experiments. sgRNAs, which were designed for each locus with the CHOPCHOP web tool (https://chopchop.cbu.uib.no/), were cloned into lentiCRISPR v.2 vector.^[^ ^59^ ^]^ Human PTPRZ1 cDNA (Gene ID: 5803) was cloned into TK‐PCDH‐CMV‐copGFP‐T2A‐Puro for overexpression experiments. Human SOX2 cDNA (Gene ID: 6657) was cloned into TK‐PCDH‐CMV‐copGFP‐T2A‐Puro for overexpression experiments. Human KLF5 cDNA (Gene ID: 688) was cloned into PCDH‐CMV‐MCS‐EF1‐RFP‐T2A‐Puro for overexpression experiments. LentiCRISPR v.2 was purchased from Addgene (no. 52961). The sequences of sgRNAs used in this study and all genotyping primers are listed in Table S2 (Supporting Information).

### Dual‐Luciferase Reporter Assay

For the luciferase assays, the sequences of the 9 cis‐regulatory elements enclosed by the three SOEs near *PTPRZ1* were obtained from ENCODE. These fragments were amplified by polymerase chain reaction (PCR) and then inserted into the upstream of minP in the pGL4.23 vector (Promega), respectively. The internal Renilla control plasmid pGL4.74 (hRluc/TK) (Promega) and the target plasmids (molar ratios: 1:20) were co‐transfected into cells using jetOPTIMUS Transfection Reagent (101000006, Polyplus) in 24‐well plates. After 48 h, enhancer activity was measured by Dual Luciferase Assay (E1910, Promega) following manufacturer's protocol. In all cases, the firefly luciferase activity was normalized to the Renilla signal and reported as a proportions relative value to measure the enhancer activity. All data were obtained from at least three replicative experiments and two‐tailed Student's t tests were performed to assess the statistically significant difference. The primers used for enhancer elements amplification are listed in Table S2 (Supporting Information).

### Cell Proliferation Assay

CCK8 assay and Colony formation assay were carried out to assess the proliferation of LUSC cells with PTPRZ1 knock out or overexpression. Cell viability was evaluated with cell counting kit‐8 (CCK8) according to instructions (US Everbright, China), H520‐control/H520 ‐PTPRZ1‐KO (8000 cells/well) were incubated in 96‐well plates for 24, 48, 96 and 120 h. HCC95‐control /HCC95 ‐PTPRZ1‐KO (1500 cells/well) were incubated in 96‐well plates for 24, 48, 72 h. At specific time points, the CCK8 solution was added into wells and the absorbance was measured at 450 nm (OD450) using a microplate reader following a 1h of incubation. Additionally, for colony formation assay, H226‐control/H226 ‐PTPRZ1‐OE (1000 cells/well) were placed at 6‐well plates and allowed to grow for 2 weeks. Subsequently, the developed colonies were fixed, stained and counted using a light microscope. All data were obtained from at least three replicative experiments and two‐tailed Student's t tests were performed to assess the statistically significant difference.

### Transwell Migration Assays

To perform Transwell migration assays, H226‐control/H226 ‐PTPRZ1‐OE cells were cultured in 6‐well plates and reached 90% confluence, H226‐control/H226 ‐PTPRZ1‐OE cells were serum‐starved for 12 h in basal medium prior to seeding. Subsequently, 150µL of serum‐free medium containing the cells (4 × 10^4^) suspension was carefully added to the upper chambers of Transwell inserts (8 µm pore size; Corning). The lower chambers were filled with complete medium containing 10% FBS as a chemoattractant. After incubation for 24 h at 37 °C in 5% CO_2_, migrated cells on the lower surface were fixed with 4% paraformaldehyde for 15 min and stained with 0.1% crystal violet for 10 min at room temperature, non‐migrated cells on the upper membrane surface were removed with cotton swabs. Cell migration was quantified by counting three random fields per insert under a phase‐contrast microscope.

### Reverse Transcription and Quantitative PCR

Total RNA was isolated with TRIzol reagent (15596018, Invitrogen) from cultured cells and was reversely transcribed into cDNA (R212, Vazyme) and then subjected to RT‐qPCR using a ChamQ Universal SYBR qPCR Master Mix (Q711, Vazyme). Expression of each gene tested in this study was normalized to that of ACTB (β‐actin). *P* value was calculated by two‐tailed Student's t test. The primers used for qPCR are listed in Table S2 (Supporting Information).

### Western Blot

Cells were lysed with RIPA buffer containing protease inhibitor cocktails (04693132001, Roche) and pipetted 20 times to break down genomic DNA. Then the extracts were mixed with loading buffer and run on a 10% SDS‐PAGE, and proteins were transferred to PVDF membrane (ISEQ00010, Millipore). After blocking with 5% non‐fat milk in TBST, the membranes were then incubated with the primary antibodies and followed by horseradish peroxidase (HRP)—linked secondary antibodies (M21002, Abmart). The signals were detected by western chemiluminescent HRP substrate (WBKLS0100, Millipore) according to manufacturer's instructions. The p63 (13109S, Cell Signaling Technology), SOX2 (2748S, Cell Signaling Technology), KLF5 (ab137676, Abcam), GRHL2 (ab271023, Abcam), PTPRZ1 (610179, BD), GAPDH (10494‐1‐AP, proteintech), pPI3K (4228T, Cell Signaling Technology), PI3K (4257T, Cell Signaling Technology) were used in this assay.

### Activation of Intracellular Signaling Pathways by Exogenous MDK

Six hundred thousand HCC95‐NC and HCC95‐KO‐PTPRZ1 cells in 2 mL 5% FBS T‐medium perdish were plated in 60‐mm dishes and 12 h later changed into serum‐free DMEM for another 12 h. Then, 200 ng mL^−1^ recombinant human MDK (450‐16‐20UG, PeproTech) was added to the cells for 15 min. The cells were harvested for protein isolation and Western blot analysis as described previously. The antibodies used were: GAPDH (10494‐1‐AP, proteintech), pPI3K (4228T, Cell Signaling Technology), PI3K (4257T, Cell Signaling Technology).

### Xenograft Studies

For xenograft KO tumor models, 1 × 10^7^ H520‐control/H520‐PTPRZ1‐KO and 1 × 10^8^ HCC95‐control/HCC95 ‐PTPRZ1‐KO  cells were harvested with 1% trypsin‐EDTA, washed and resuspended in sterile PBS. Cells were implanted into the right flank of 6–8 weeks old female NCG mice (n = 6 per group). Following cell inoculation, mice were monitored weekly for body weight and tumor volume (measured by caliper) until palpable tumors were detected. Once tumor growth was established, measurements were performed twice weekly, where tumor volume was calculated by the formula (length × width2)/2. In each case, when tumor volume reached >2000 mm^3^ it took the decision that the mice had reached endpoint criteria and were then scheduled for euthanasia. Since euthanasia scheduling typically took 1–4 days, tumors inevitably continued to grow rapidly, few reaching greater size (>2000 mm^3^) at the time of sacrifice.

### Immunohistochemistry (IHC)

The expression of PTPRZ1 and Ki‐67 in human and mouse tumor tissues was evaluated by immunohistochemistry (IHC). Formalin‐fixed, paraffin‐embedded (FFPE) tumor sections were prepared and stained with a fully automated IHC Ventana Benchmark XT stainer (Ventana Medical Systems, Inc., AZ, USA). The following primary antibodies were used: PTPRZ1 (55125‐1‐AP, Proteintech, 1:100), and Ki‐67 (ab16667, Abcam). To ensure technical consistency and minimize errors, both negative and positive controls were included in each assay.

The IHC score ranged from 0 to 12 is the product of multiplying the positive‐cell‐proportion score (1‐4) and the staining‐intensity score (0‐3). For staining intensity, the scoring was as follows: 0 for no staining, 1 for weak staining, 2 for moderate staining, and 3 for strong staining. The positive‐cell‐proportion score was determined based on the following thresholds: less than 25% positive cells scored 1; 26–50% positive cells scored 2; 51–75% positive cells scored 3; and greater than 75% positive cells scored 4.

### Whole‐Genome Sequencing

Genomic DNA was extracted from isogenic HCC95 control and PTPRZ1‐KO cell lines using the TIANamp Genomic DNA Kit (TIANGEN), with quality verified by Quibt 4 and electrophoresis. Genomic DNA were sonicated to generate chromatin fragment on a Covaris M220 Focused‐ultrasonicator (Insert, microTUBE 130 µL; Temperature, 7 °C; Peak Incident Power, 50W; Duty Factor, 20%; Treatment Time, 65s). Libraries were prepared from 4 µg fragmented DNA (≈350 bp) with the Illumina TruSeq DNA PCR‐Free Kit and sequenced on NovaSeq X Plus (PE150, 30× coverage).

Sequence read quality was assessed using FastQC (version 0.11.9), adapters were trimmed using trim_galore (version 0.6.10). Reads passing Illumina's chastity filter were mapped to the human reference genome (hg38) using BWA (version 0.7.17). The Genome Analysis Toolkit (GATK, version 4.2.6.1) was used to call variants with the default parameters of GATK Best Practices. The resulting variant call format (VCF) files were filtered to retain variants with DP > 9 and AF > 0.1 using bcftools (version 1.21). Indels were considered “near” low complexity regions if any position within 10 bp or at least one third of positions within 50 bp were masked by repetitive intervals. Subsequently, sites where each DNA‐edited cell line showed either a heterozygous SNP or an indel call that was not observed in the unedited cell line were considered as mutations if at least 30% of reads in the edited cell line contained the mutation, no allelic content derived from the mutation was present in the unedited line, and if the candidate mutation had not previously been observed in the dbSNP database.

### Statistical Analysis

All data were presented as the mean ± standard error of the mean from at least three independent experiments. Statistical analyses were performed using GraphPad Prism 9.0 software. The significance of differences between groups was estimated using the Student's t‐test. The probability of survival was estimated using the Kaplan–Meier method, and differences between groups were evaluated using the log‐rank test. All statistical analyses were performed using two‐tailed p values, and the statistical significance threshold was set at 0.05 if not explicitly mentioned (* *p* < 0.05, ** *p* < 0.01, *** p < 0.001 and **** *p* < 0.0001).

### Data Availability Statement

The LUSC ATAC‐seq peak data are downloaded from https://api.gdc.cancer.gov/data/d28f95fc‐af3b‐497d‐806b‐eb0625d6c831, the normal ATAC‐seq peak data are downloaded from ENCODE with ID ENCFF818EYD. LUSC scRNA‐seq data from previous published studies, among them, 15 samples are publicly available at BioStudies (https://www.ebi.ac.uk/biostudies/) with accession numbers E‐MTAB‐13526, the other two samples are available in Science Data Bank (ScienceDB, https://www.scidb.cn/en) by visiting 10.57760/sciencedb.02028. The HiChIP publicly available data used in this study were downloaded from the Gene Expression Omnibus (GEO) series GSE166234 (for NCI‐H520 cell). Due to privacy considerations of human genetic information, the raw datasets (ChIP‐seq, RNA‐seq, scATAC‐seq) will be made available through a formal data request process following institutional guidelines. The raw reads of ChIP‐seq, RNA‐seq of all cell lines are deposited in GEO, with an accession no. GSE293734. The data is also backed up in China National Center for Bioinformation under accession no. HRA011029, which can be reached through the URL: https://ngdc.cncb.ac.cn/gsa‐human/s/6qIF6dzx. All primer sequences used in this study are available in Table S2 (Supporting Information).

## Conflict of Interest

The authors declare no conflict of interest.

## Author Contributions

Y.N., J.W., Y.Z., H.L., and Z.J. contributed equally to this work. J.Y. and W.L. conceived the project. Y.N., J.W., H.L., Z.J., X.Y., X.S., X.W., B.W., and J.Y. carried out experiments, and Y.Z. and J.Y. performed data analysis. J.Y., W.L., J.W., Y.Z., and Y.N. wrote the manuscript.

## Supporting information



Supporting Information

Supplemental Table S1

Supplemental Table S2

## Data Availability

The data that support the findings of this study are available on request from the corresponding author. The data including patients' genetic information are not publicly available due to privacy or ethical restrictions.
