# Supplementary material for: Enhancer Reprogramming Reveals the Tumorigenic Role of PTPRZ1 in Lung Squamous Cell Carcinoma
Source: Adv Sci (Weinh). 2025 Sep 3;12(44):e09344. doi: 10.1002/advs.202509344 (PMC12667478; doi:10.1002/advs.202509344)
Supplement: Supplementary file 1 — Supporting Information [file ADVS-12-e09344-s001.docx]

**Supplementary** **Figure Legends**

**
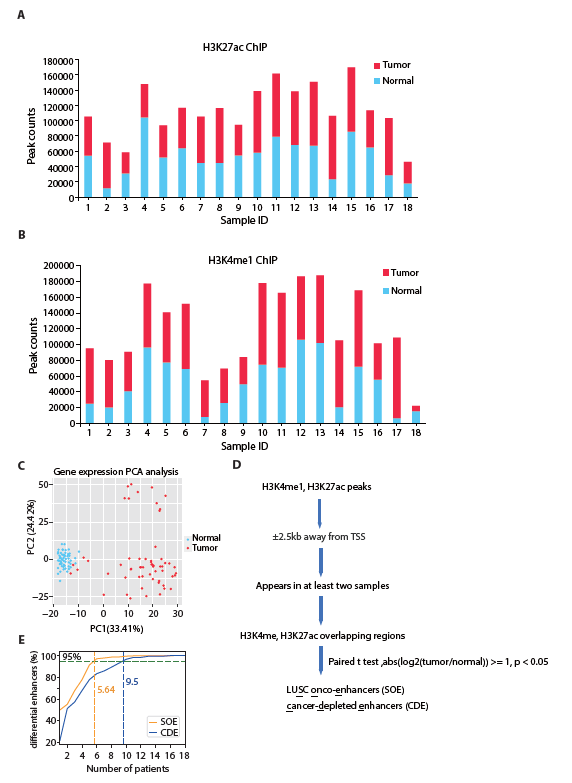
**

**Figure S1. Quality Control and Identification of Differential Enhancers in LUSC.**

**(A**, **B)** Quality assessment of chromatin profiling data. Display of H3K27ac (a) or H3K4me1 (b) ChIP-seq peak distributions across 18 paired tumor-normal samples. Total sequencing reads per sample reflect comparable coverage between tumor (red) and normal (blue) specimens.

**(C)** PCA analysis of RNA-seq data from 59 paired tumor-normal samples reveals clear separation. Tumor samples (blue) cluster distinctly from normal tissues (red). Global expression divergence between normal and tumor tissues was analysed with MANOVA, yielding significant results (Pillai's trace = 0.922, p < 0.001, partial η² = 0.119).

**(D)** Flow chart outlining the process for defining differential enhancers. This includes steps for identifying significant enhancer activity and criteria for classification.

**(E)** Saturation analysis illustrating the percentage of newly identified SOEs (orange) and CDEs (blue) compared with the total number of significant enhancers, as more tumor or normal samples are analyzed. Two vertical dashed lines mark the recurrence of SOEs and CDEs upon reaching the cut-off at 95% (aqua dashed line) of significant percentage.


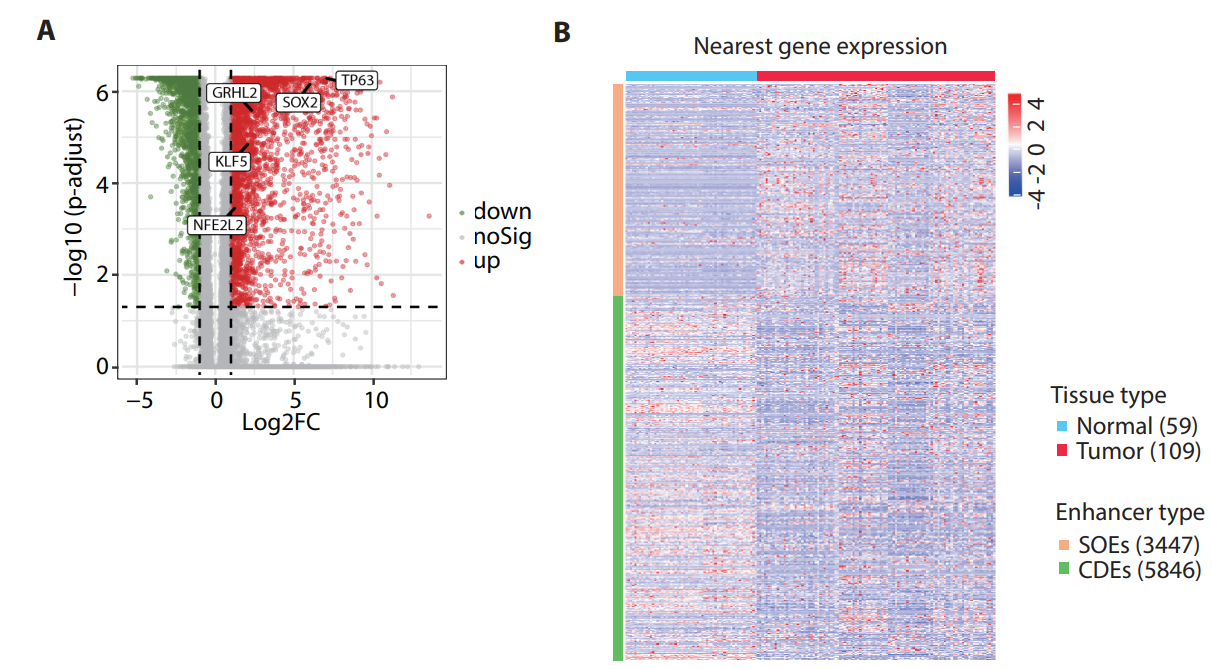


**Figure S2. Transcriptional Landscapes Distinguish LUSC Tumors from Normal Tissues.**

**(A)** Volcano plot illustrating mRNA enrichment in 59 paired tumor-normal samples. Upregulated mRNAs are marked in red dots, while downregulated mRNAs are highlighted in green.

**(B)** Heatmap depicting nearest gene expression profiles of SOEs and CDEs among tumor (n=109) and native (n=59) tissues from LUSC patients.


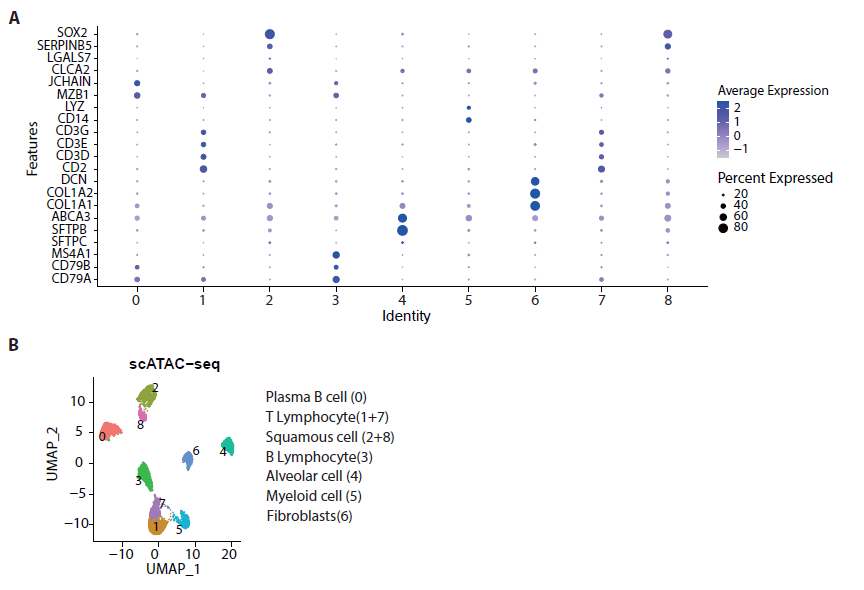


**Figure S3. Single-cell chromatin landscapes define the LUSC tumor ecosystem.**

**(A)** Cell-type annotation by chromatin accessibility. Dot plot shows cell clusters (0-8) annotated based on chromatin accessibility in promoters of marker genes (e.g., SOX2) from scATAC-seq analysis (n=2 tumor samples).

**(B)** UMAP visualization of scATAC-seq data (n=2 tumors) confirming cell type classification through chromatin accessibility patterns.

**
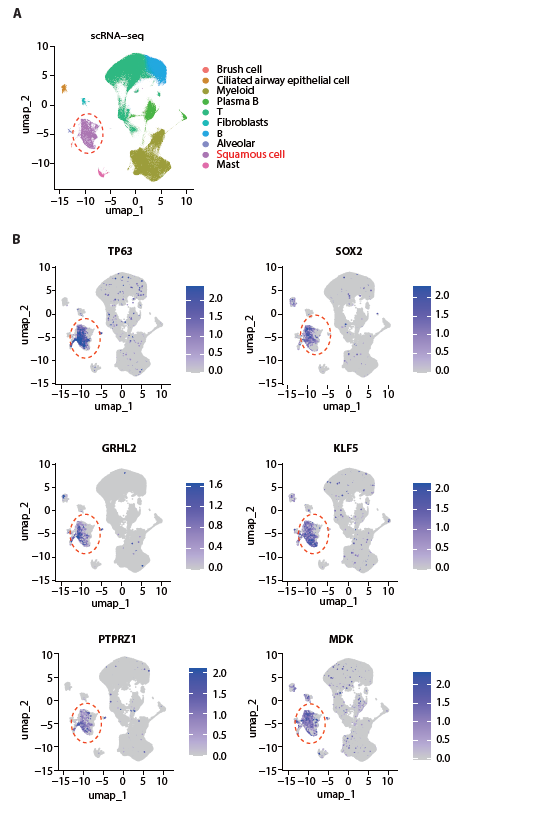
**

**Figure S4. Single-cell transcriptional profiling of LUSC tumors reveals squamous-specific expression patterns.**

**(A)** UMAP projection of scRNA-seq data from 17 LUSC tumors with detailed cell type annotations (dotted lines represent Squamous cells).

**(B)** Expression patterns of key regulatory genes (TP63, SOX2, GRHL2, KLF5, PTPRZ1, MDK) across tumor cell clusters (dotted lines represent Squamous cells).

**
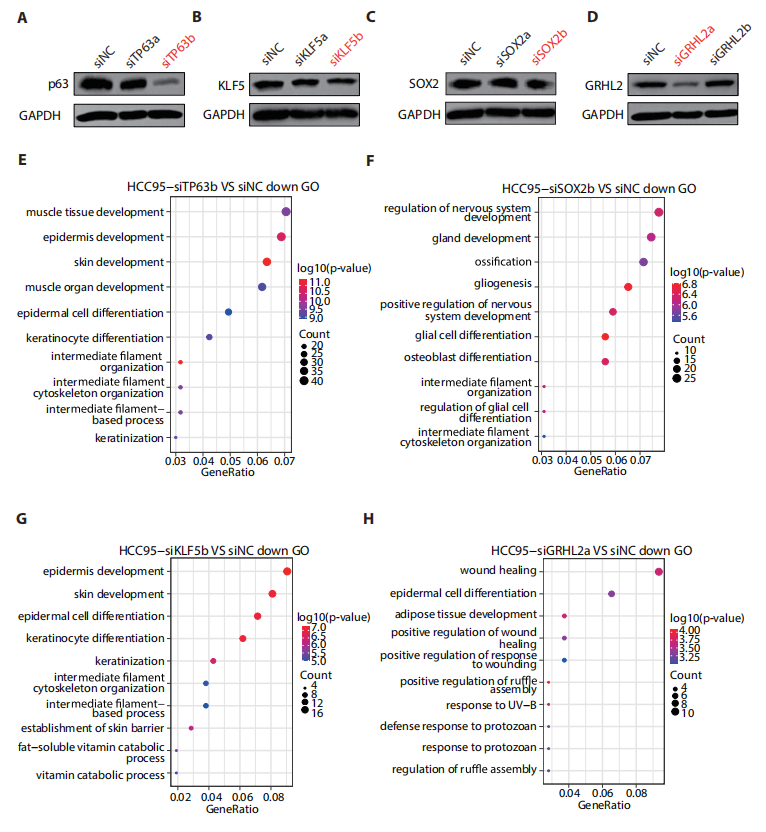
**

**Figure S5. Transcriptional network analysis following oncogenic TF knockdown in HCC95 cells.**

**(A-D)** Western blot analysis of protein expression in HCC95 cells 72 hours post-transfection with TP63 (a), KLF5 (b), SOX2 (b), GRHL2 (d) siRNAs, or Negative control siRNA (siNC). GAPDH was used as loading control.

**(E-H)** Bubble plot showing enriched GO biological process terms (FDR < 0.05) for downregulated genes in HCC95 cells transfected with TF-targeting siRNA compared to negative control.


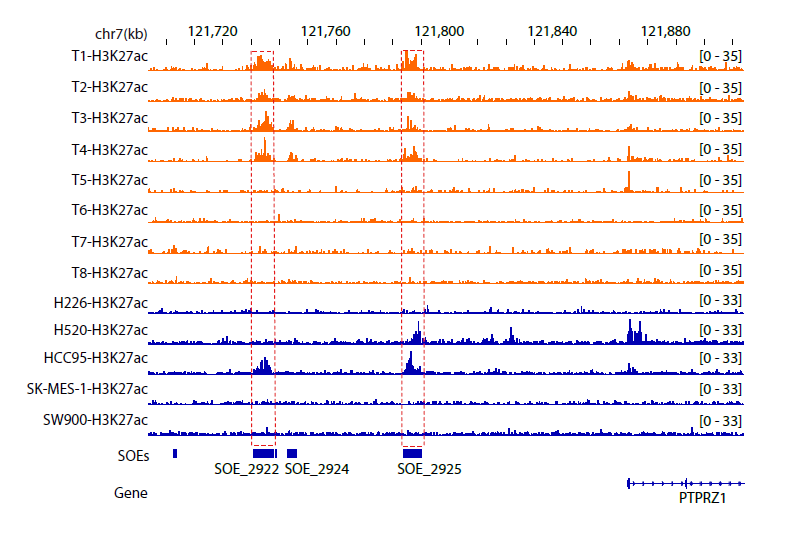


**Figure S6.** **Epigenomic landscape of the *PTPRZ1* Locus.**

Top: H3K27ac ChIP-seq profiles in LUSC tumor tissues. The tumor sample T1–T4 epigenetic pattern is observed in 15 of 18 samples, while the T5–T8 pattern occurs in 5 samples. Others not shown. Bottom: H3K27ac ChIP-seq profiles in LUSC cell lines (H226, H520, HCC95, SK-MES-1, SW900). The solid blue line at the bottom indicates the identified SOE.


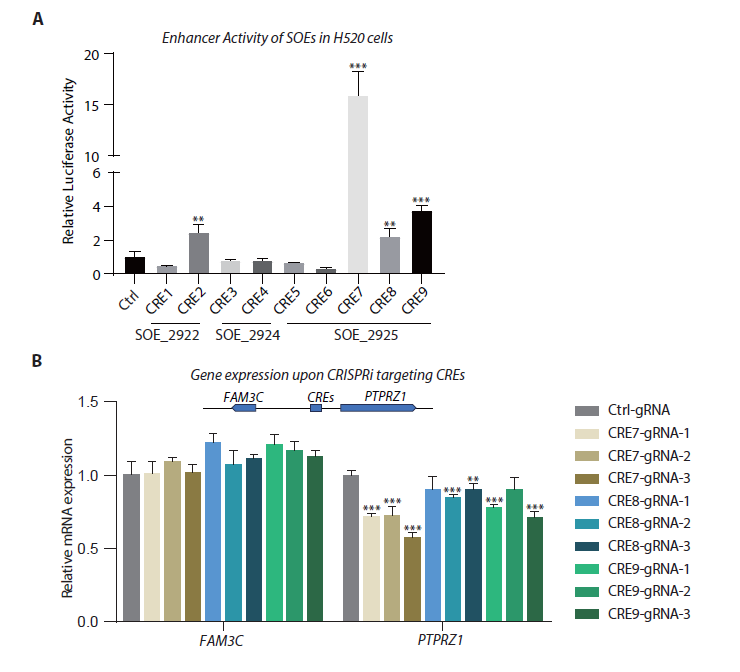


**Figure S7. Functional validation of SOEs in H520 cells.**

**(A)** Luciferase reporter assays in H520 cells demonstrate significant enhancer activity at SOEs (SOE_2922/2924/2925). Relative luminescence measured 48h post-transfection (Firefly/Renilla ratio, normalized to empty vector control) (Data are shown as the mean ± s.e.m., n=4 , *P* values were assessed using two-tailed Student’s t tests, ***P*<0.01, ****P*<0.001).

**(B)** PTPRZ1 expression in HCC95 cells was assessed by RT-PCR following CRISPRi targeting of SOE_2922 and SOE_2925. gRNAs were designed against CRE7, CRE8, and CRE9 in SOE_2925. Three highest-scoring gRNAs per CRE were selected for CRISPRi. FAM3C, located upstream of these CREs, served as the control. (normalized to ACTB; Data are shown as the mean ± s.e.m., n=4; P values were assessed using two-tailed Student’s t tests; **P<0.01, ***P<0.001).

**
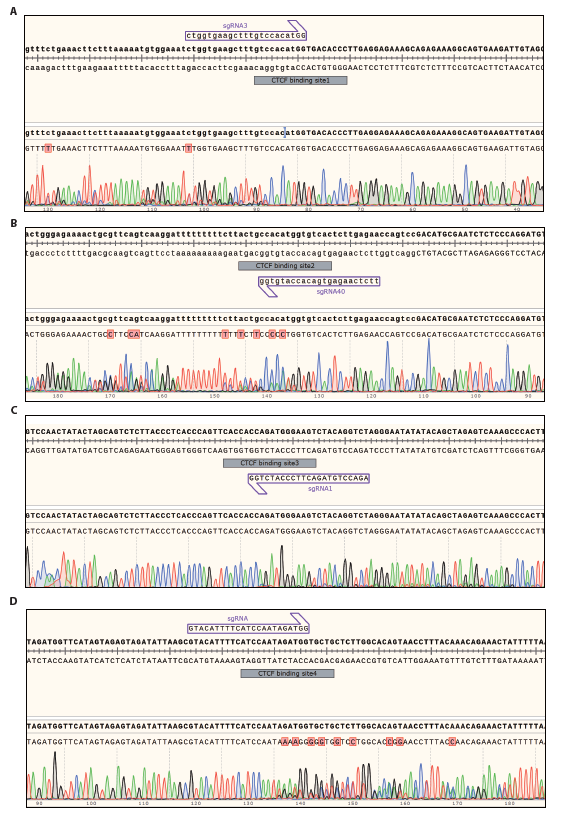
**

**Figure S8. CRISPR-Cas9 targeting strategy for PTPRZ1 regulatory elements.**

sgRNA sequence designed to target the CTCF binding sites in the regulatory region of PTPRZ1 (sites 1-4), showing the locations of 4 sgRNAs and Sanger-sequencing of edited H520 cells, respectively.

**
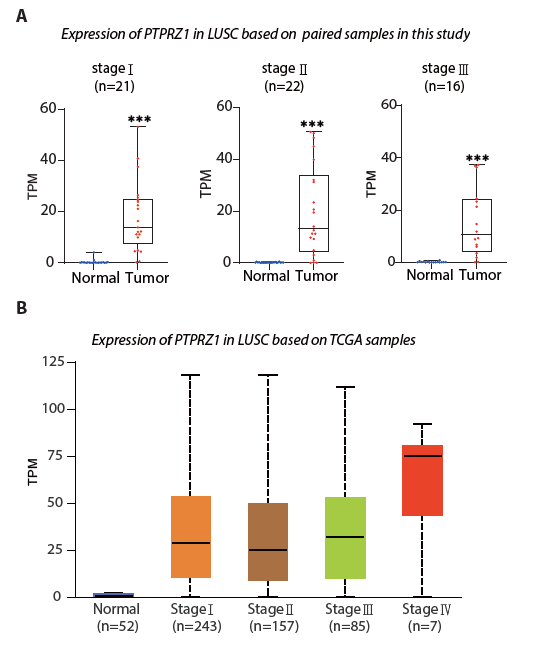
**

**Figure S9. Clinical relevance of PTPRZ1 expression across LUSC progression stages.**

**(A)** Paired analysis of PTPRZ1 mRNA expression (RNA-seq TPM) in LUSC cohort (Stage I-III). *P* values were assessed using two-tailed Student’s t tests, ****P*<0.001.

**(B)** PTPRZ1 expression data in LUSC from TCGA samples (Normal and stages I-IV).


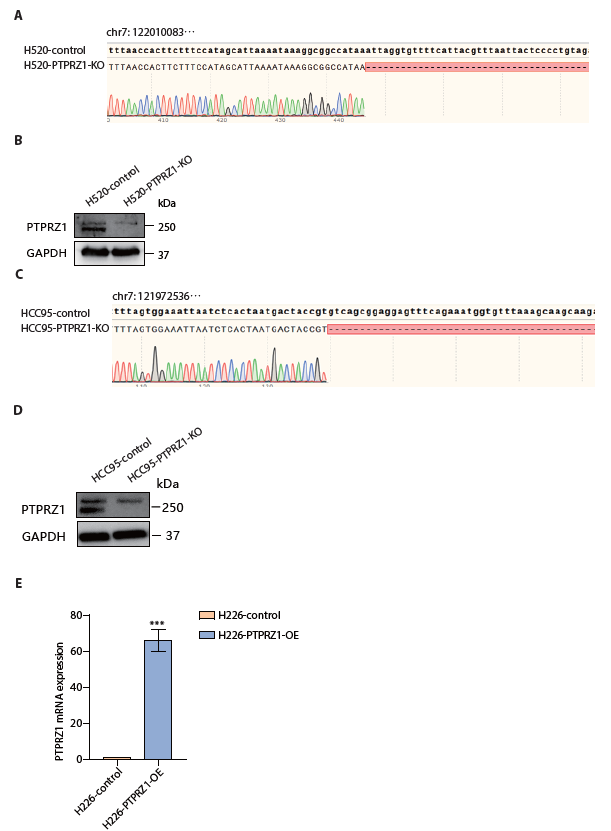


**Figure S10. Validation of PTPRZ1 knockout and overexpression in LUSC cell lines.**

**(A, C)** Sequence alignment of PTPRZ1-KO H520 (a) and HCC95 (c) cells with wild-type counterparts, confirmed by Sanger sequencing.

**(B, D)** PTPRZ1 expression levels detected by WB in H520 (b) and HCC95 (d) knockout cell lines.

**(E)** RT-qPCR analysis showing increased PTPRZ1 expression in H226 cells post-overexpression (Data are shown as the mean ± s.e.m., n=3, *P* values were assessed using two-tailed Student’s t tests, ****P*<0.001).

**
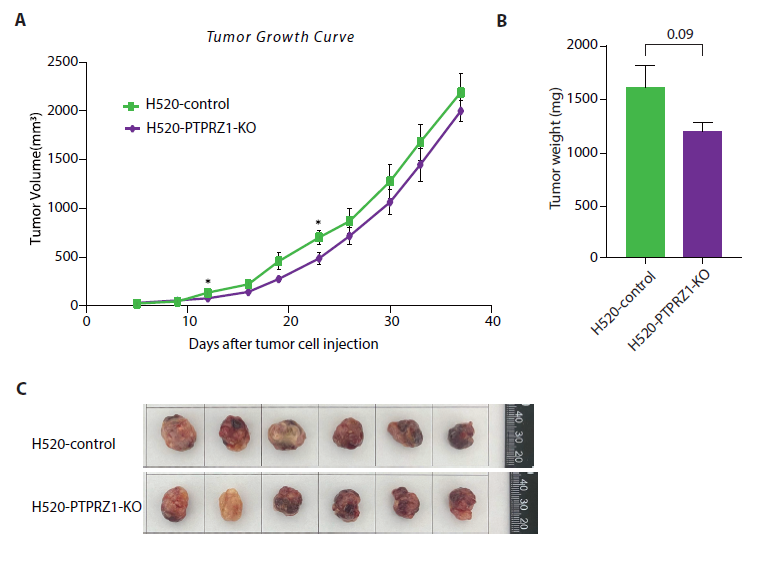
**

**Figure S11. PTPRZ1 is essential for LUSC tumorigenesis *in vivo*.**

Tumor size, volumes, and weight between control and PTPRZ1 knockout in H520 cells in xenograft experiments. PTPRZ1-KO group shows non-significant reduced tumor growth compared to controls (Student’s t tests, n=6, *P*=0.09).

**
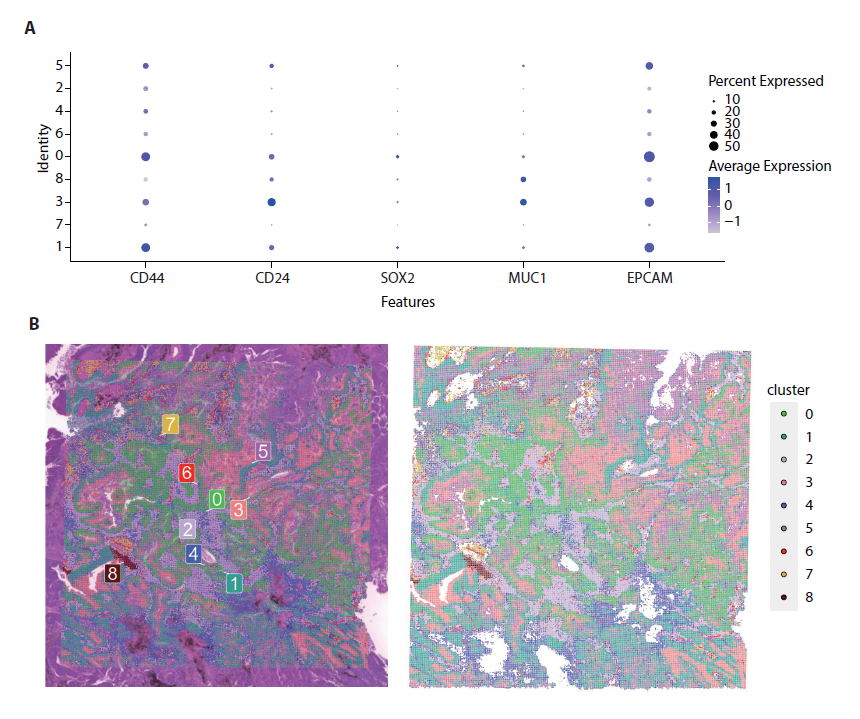
**

**Figure S12. Spatial transcriptomic profiling of the LUSC tumor microenvironment.**

**(A)** Cell-type annotation by spatial transcriptomic profiles. Dot plot shows clusters based on marker gene expression using 10X Visium HD.

**(B)** Visualization of clusters based on spatial location, with two image variations for clarity.

**
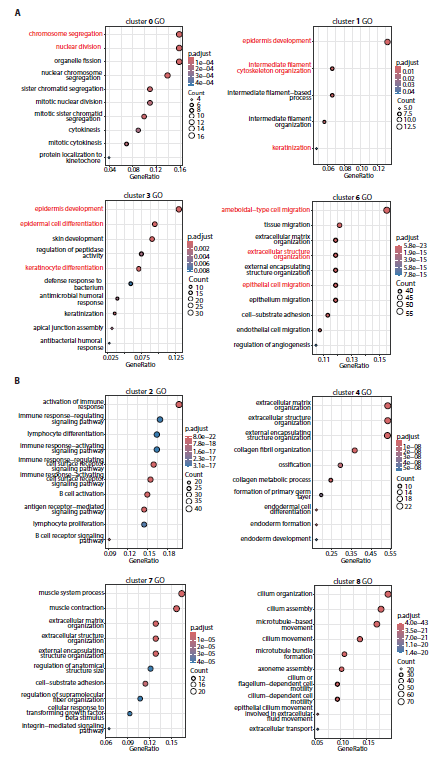
**

**Figure S13. GO analysis of different cell clusters in LUSC microenvironment.**

**(A-B)** Bubble plot showing enriched GO biological process terms (FDR < 0.05) for cluster-specific genes expressed in tumor-like clusters (a) and other clusters (b) within the microenvironment.

**Supplementary Table legends**

**Supplementary Table 1. Data sets used in this study.**

**A**, Summary of the clinical data for 109 patients in this study.

**B**, SOEs peaks obtained by differential enhancer analysis between tumor and adjacent tissues.

**C**, CDEs peaks obtained by differential enhancer analysis between tumor and adjacent tissues.

**D**, Squamous cell cluster specific ATAC peaks.

**E**, Genes within ± 0.2Mb peaks where the SOE peak and the squamous cell cluster-specific ATAC peak intersect.

**F**, Tumor specifically upregulated genes obtained by bulk RNA-seq.

**G**, Squamous cell cluster specifically expressed genes obtained by scRNA-seq.

**H**, master TF co-expressed genes. Please note that when screening, *P* value < 0.05 and r > 0.5 are required.

**I,** Summary of whole-genome sequencing analysis results of PTPRZ1-KO cells.

**Supplementary Table 2. Primers used in this study.**
